# Structural basis of membrane engagement and polyreactivity control in HIV-1 MPER broadly neutralizing antibodies

**DOI:** 10.1073/pnas.2609827123

**Published:** 2026-07-14

**Authors:** So Yeon Cho, Kimmo Rantalainen, Gabriel Ozorowski, Danny Lu, Ryan Tingle, Wen-Hsin Lee, Andrew B. Ward, William R. Schief, Ian A. Wilson

**Affiliations:** ^a^ Department of Integrative Structural and Computational Biology, The Scripps Research Institute, La Jolla, CA 92037; ^b^ International AIDS Vaccine Initiative Neutralizing Antibody Center, The Scripps Research Institute, La Jolla, CA 92037; ^c^ Center for Human Immunodeficiency Virus/Acquired Immunodeficiency Syndrome Vaccine Immunology and Immunogen Discovery, The Scripps Research Institute, La Jolla, CA 92037; ^d^ Department of Immunology and Microbiology, The Scripps Research Institute, La Jolla, CA 92037; ^e^Moderna Inc., Cambridge, MA 02142

**Keywords:** HIV-1 MPER broadly neutralizing antibodies, lipid–antibody interactions, X-ray crystallography, electron microscopy

## Abstract

The membrane-proximal external region (MPER) of HIV-1 Env represents a critical target for broadly neutralizing antibodies (bnAbs) due to its conservation and functional importance. However, MPER-targeting bnAbs recognize composite epitopes comprising peptide and viral membrane lipid components, creating an inherent tension between viral neutralization efficacy and polyreactivity. 10E8-class antibodies exhibit high neutralization potency with low polyreactivity, whereas 4E10-class antibodies show comparably broad neutralization but higher polyreactivity, underscoring the need to understand the structural basis of this distinction. We therefore determined crystal structures of DH511.1 (memory B cell–derived), DH511.12P (plasma cell-derived), and VRC42.01 in complex with MPER peptide and phosphatidic acid, along with a cryo-EM reconstruction of DH511.2 bound to membrane-embedded Env. Through integrative analysis taking into account previously determined structures of other MPER bnAbs, we reveal two distinct lipid recognition strategies. Groove-mediated binders, including 10E8 and DH511, engage lipids through antibody–membrane interface grooves with distinct geometries and angular approaches to the membrane. In contrast, heavy chain-mediated binders, including 4E10, PGZL1, and VRC42, utilize positively charged CDR H1 patches for direct lipid headgroup recognition. Importantly, DH511 lineage members exhibited differential cardiolipin polyreactivity linked to their maturation stage. PGZL1 and VRC42.01 employ weaker positive patches at lipid-binding sites than 4E10, and PGZL1 additionally introduces a CDR H3-mediated negative patch that creates electrostatic repulsion with negatively charged lipid headgroups, thereby limiting nonspecific interactions. These findings provide a structural framework for understanding how MPER bnAbs balance lipid binding with specificity and inform immunogen design for inducing safe and effective neutralizing responses.

## Significance

Broadly neutralizing antibodies (bnAbs) targeting the HIV-1 membrane-proximal external region (MPER) engage both viral protein and membrane lipids, but this requirement carries a risk of unwanted polyreactivity. Here, crystal structures of DH511- and VRC42-lineage antibodies, combined with electron microscopy, reveal two distinct mechanistic strategies for lipid recognition by MPER bnAbs. Lipid-binding analyses further show how antibody maturation can modulate polyreactivity within a single lineage. Notably, DH511 lineage antibodies with high neutralization breadth comparable to 10E8 suppress polyreactivity through structural and genetic tuning, whereas VRC42 and PGZL1 mitigate the high polyreactivity characteristic of 4E10 through electrostatic modulation. These findings explain how effective MPER bnAbs balance specific membrane engagement while reducing polyreactivity that can aid in next-generation HIV-1 MPER vaccine design.

The development of an effective vaccine to prevent HIV type 1 (HIV-1) infection remains a major global public health challenge (1–3). The HIV-1 envelope glycoprotein (Env) serves as the primary target for broadly neutralizing antibodies (bnAbs), with the membrane-proximal external region (MPER) of Env gp41 representing a particularly attractive target due to its essential role in viral fusion and high sequence conservation (4, 5). MPER-targeting bnAbs demonstrate exceptional neutralizing breadth, with some capable of neutralizing over 98% of tested HIV-1 isolates (6–8).

Among MPER-directed bnAbs, 2F5 targets the N-terminal region of MPER (residues 659 to 671) (9–13). In contrast, antibodies with greater neutralization efficacy, including 10E8, DH511, 4E10, PGZL1, and VRC42, recognize the MPER C-terminal helical region (residues 671 to 683) adjacent to the transmembrane domain (7, 8, 14–17). LN01 recognizes an extended, semiconcealed MPER epitope (residues 671 to 711) that spans into the transmembrane region, rendering it partially occluded by the viral membrane (*SI Appendix*, Table S1) (18). Due to the membrane-proximal nature of the MPER region, these bnAbs are characterized by a long hydrophobic complementarity-determining region (CDR) H3 (17 to 24 amino acids) with aromatic residues at the tip for membrane insertion (7, 18–22). The complete epitope of MPER bnAbs has been shown to be composite, including both MPER peptide and viral membrane lipid components (5, 23–25).

The ability of antibodies to interact with lipid bilayers is crucial for MPER epitope recognition and neutralization (6, 26–29), but simultaneously poses autoimmune risks through reaction with host membranes (30–32). Antibodies such as 4E10 and 2F5 exhibit strong polyreactivity and autoreactivity to various host membrane components, limiting their therapeutic and vaccine development potential (31, 33, 34). Therefore, understanding lipid-binding mechanisms while minimizing polyreactivity is crucial for HIV-1 vaccine design (35, 36).

Among MPER bnAbs, 10E8 and the DH511 lineage share the V_H_3-15 germline gene, while 4E10, PGZL1, and VRC42.01 share V_H_1-69, V_K_3-20, and D3-10 germline genes (*SI Appendix*, Table S1) (37). 10E8 shows the highest neutralizing potency with low polyreactivity (8, 23, 27, 38). The DH511 lineage comprises memory B cell–derived members (DH511.1–DH511.6) and plasma cell-derived members (DH511.7P-DH511.12P, with “P” indicating plasma origin), several of which exhibit similar MPER recognition to 10E8 (16). These lineage members share a polyreactive unmutated common ancestor (UCA) yet exhibit differential neutralization potency and poly/autoreactivity profiles. The memory B cell repertoire reflects an individual’s cumulative immune history (39), whereas plasma antibodies are predominantly contributed by long- and short-lived plasma cells (40). Thus, cellular origin and the extent of affinity maturation may shape the functional properties of each lineage member. The V_H_1-69/V_K_3-20/D3-10-encoded antibodies induce a characteristic 3_10_ helical turn in MPER residues 672 to 674 upon binding (7, 17, 41, 42). Both 4E10 and PGZL1 employ heavy chain-mediated lipid binding through CDR H1 interactions with lipid headgroups and CDR H3 engagement with hydrophobic tails (42). While VRC42.01 demonstrates binding to sphingolipids and phospholipids similar to 4E10 (17), its precise lipid-binding sites remain structurally undefined. Both PGZL1 and VRC42.01 are characterized by shorter CDR H3 and reduced polyreactivity compared to 4E10, while maintaining comparable neutralizing activities (7, 17). Notably, VRC42.UCA displays little polyreactivity, providing a promising framework for vaccine-induced responses (17).

Previous structural studies have identified MPER-binding modes and lipid complex structures with several antibodies (7, 8, 12, 14–18, 20, 24, 43–47). However, the lipid-binding sites of DH511 and VRC42 lineages have not been clearly defined. Moreover, DH511 and VRC42 originate from multiclonal lineages, but how somatic maturation or clonal origin affects antibody polyreactivity or lipid binding have not been elucidated.

To address these knowledge gaps, we present structural and functional comparisons of MPER and membrane recognition by MPER bnAbs. We determined high-resolution crystal structures of memory B cell–derived DH511.1 and plasma cell-derived DH511.12P from the DH511 lineage, and VRC42.01 from the VRC42 lineage, in complex with MPER peptide and lipids, directly visualizing their lipid-binding sites. We further obtained a cryo-EM reconstruction of memory B cell–derived DH511.2 bound to membrane-embedded Env trimers, revealing how the antibody recognizes MPER and the viral membrane in a native-like context. Through integrative analysis with existing lipid-bound and membrane-embedded Fab structures, combined with lipid-binding analyses and mutagenesis studies, we elucidated the molecular mechanisms of effective MPER- and membrane- targeting and identify the key structural determinants regulating polyreactivity. This study provides a foundation for developing next-generation HIV-1 vaccine immunogens capable of inducing broadly neutralizing responses against this conserved yet challenging MPER epitope.

## Results

### Lipid-Binding Profiles of HIV-1 gp41 MPER bnAbs.

**Fig. 1.**
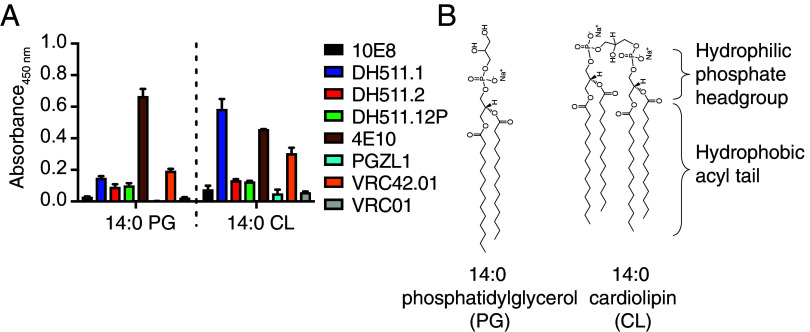
Lipid-binding profiles of HIV-1 MPER bnAbs. (*A*) Fab binding to 14:0 PG (dimyristoyl phosphatidylglycerol) and 14:0 CL (dimyristoyl cardiolipin) as measured by ELISA to assess intrinsic polyreactivity profiles. The VRC01 Fab was used as a negative control. Data are representative of three independent experiments with consistent results. (*B*) Two-dimensional schematic representation of 14:0 PG and 14:0 CL.

To assess the intrinsic polyreactivity profiles of MPER bnAbs, we evaluated Fab binding to phosphatidylglycerol (PG), which is enriched in HIV-1 membranes during viral budding (48–50), and cardiolipin (CL), a mitochondrial lipid marker for autoreactivity (51) (Fig. 1). In our assay, consistent with established reports (7, 17, 52), 4E10 and VRC42.01 displayed marked binding to both PG and CL, although VRC42.01 binding was less than that of 4E10, while 10E8 and PGZL1 showed negligible binding to either lipid. Of interest, we observed differential CL-binding profiles within DH511 lineage members. All DH511 members tested showed weak PG binding. However, DH511.1 displayed markedly increased CL binding that exceeded even 4E10, whereas DH511.2 and DH511.12P maintained similarly weak reactivity to both lipids. Given that the DH511 UCA exhibits inherent polyreactivity and that DH511.2 and DH511.12P demonstrate higher neutralization potency than DH511.1 (16), the pronounced CL binding of DH511.1 suggests that certain memory B cell–derived clones may retain the polyreactivity characteristics of their germline predecessors.

### Crystal Structures of DH511.1, DH511.12P, and VRC42.01 in Complex With MPER Reveal Lipid-Binding Sites.

**Fig. 2.**
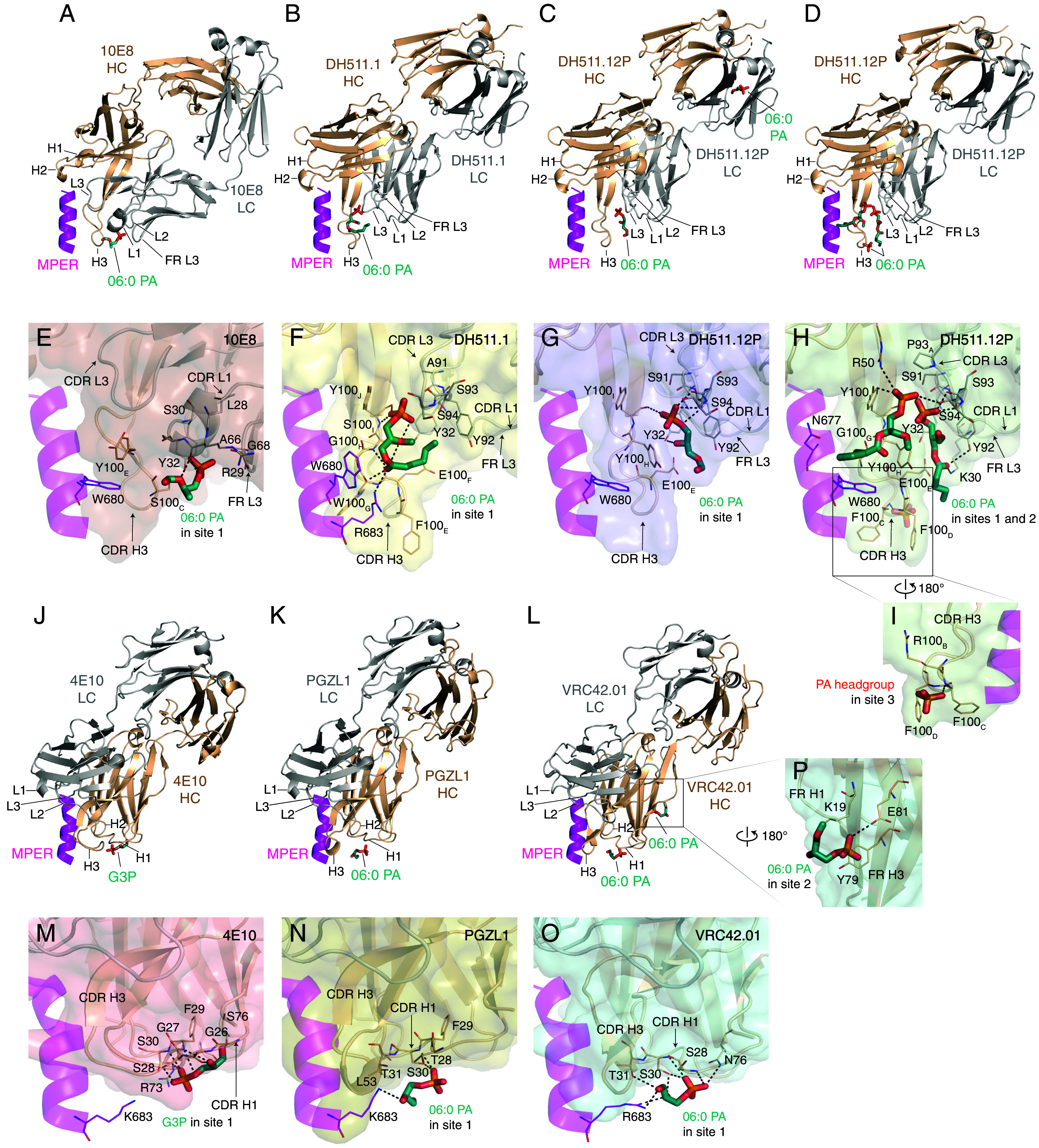
Crystal structures of HIV-1 MPER bnAbs in complex with MPER peptide and lipids. (*A–D*) Overall structures of MPER- and lipid-bound Fabs shown with MPER oriented perpendicular to the membrane include 10E8 (*A*; PDB ID: 5T80), DH511.1 (*B*), DH511.12P in single-lipid form (*C*), and DH511.12P in double-lipid form (*D*). Heavy and light chains are depicted in beige and gray, respectively. MPER peptides are shown in magenta, and lipid molecules are rendered as teal sticks with orange phosphate headgroups. (*E–I*) Detailed views of lipid-binding interfaces in the structures shown in (*A–D*). Fab and MPER residues at the lipid-binding interface are shown as sticks and labels. Fab chains are depicted as ribbons (beige and gray for heavy and light chains) with antibody-specific surface coloring, and MPER peptides are shown as magenta ribbons. Kabat numbering is used throughout for antibody residues. (*J–L*) Overall structures of 4E10 (*J*; PDB ID: 4XC1), PGZL1 (*K*; PDB ID: 6O3J), and VRC42.01 (*L*), displayed as in (*A–D*). (*M–P*) Detailed views of lipid-binding interfaces in the structures shown in (*J–L*), displayed as in (*E–I*).

While the lipid-binding sites of the MPER bnAbs, 10E8, 4E10, and PGZL1, have been structurally characterized (7, 27, 42), those of the DH511 and VRC42 lineages remained undefined. For comprehensive analysis of lipid binding across these antibodies, we performed cocrystallization of DH511.1, DH511.12P, and VRC42.01 Fabs with an MPER peptide (residues 671 to 683_,_ HxB2 numbering) and dihexanoyl phosphatidic acid (06:0 PA), yielding multiple crystal forms with variable lipid occupancy (Fig. 2 and *SI Appendix*, Fig. S1 and Table S2).

DH511.1 crystallization produced two forms (Fig. 2*B* and *SI Appendix*, Fig. S1*A* and Table S2), a lipid-bound structure at 2.06 Å resolution (space group *P*2_1_2_1_2_1_) containing Fab, MPER, and a single 06:0 PA molecule (DH511.1-MPER-PA), and an unliganded structure at 1.66 Å resolution (space group *P*2_1_) lacking ordered electron density for MPER and PA despite their presence during crystallization (DH511.1_unliganded_) (*SI Appendix*, Fig. S2*B* and Table S2). This unliganded form contained a phosphate ion at a position distinct from the PA-binding site observed in the DH511.1-MPER-PA structure, likely from the crystallization buffer (*SI Appendix*, Fig. S2*B* and Table S2). The DH511.1-MPER-PA structure showed high structural similarity to both MPER- and lipid-unbound forms over 219 Cα atoms of the Fab variable regions with root-mean-square deviations (RMSD) of 0.37 Å compared to the DH511.1_unliganded_ structure and 0.38 Å with the lipid-free DH511.1-MPER complex (PDB ID: 5U3J)] (*SI Appendix*, Fig. S2*B*).

DH511.12P crystallization also yielded two crystal forms with different lipid occupancy (Fig. 2 *C* and *D* and *SI Appendix*, Fig. S1 *B* and *C* and Table S2). The *P*2_1_ form (2.21 Å resolution) contained two DH511.12P-MPER-lipid complexes per asymmetric unit, each harboring a single 06:0 PA molecule at the CDR H3-CDR L3 interface (single-lipid form; DH511.12P-MPER-PA_one_), which is considered biologically relevant because of its proximity to the MPER epitope within the lipid-rich viral membrane environment (Fig. 2*C* and *SI Appendix*, Fig. S1*B* and Table S2). The CDR H3-CDR L3-associated region is similar to that observed in the DH511.1-MPER-PA structure and termed lipid-binding site 1. One complex in the asymmetric unit additionally bound PA in the constant light-chain region, which was considered nonbiologically relevant.

The *P*3_1_21 form (1.97 Å resolution) of DH511.12P revealed the double-lipid form (DH511.12P-MPER-PA_two_) with two PA molecules in the CDR H3-CDR L3 region (Fig. 2*D* and *SI Appendix*, Fig. S1*C* and Table S2). Although the second PA molecule appeared to be stabilized by crystal packing, this lipid-binding site 2 features hydrogen bond networks for lipid head-anchoring and hydrophobic interactions for tail accommodation, providing an appropriate biophysical environment for lipid engagement. Additional electron density consistent with a PA phosphate headgroup was detected on the opposite face of the CDR H3 loop tip, suggesting a potential lipid-binding site 3 (Fig. 2*D* and *SI Appendix*, Fig. S1*F*). The single- and double-lipid forms were highly similar (0.26 Å RMSD), as were comparisons to lipid-free DH511.12P-MPER complex (PDB ID: 5U3N; 0.43 to 0.45 Å RMSD) (*SI Appendix*, Fig. S2*C*). Combined with the structural similarity between lipid-bound and -unbound 10E8-MPER complexes (0.34 Å RMSD) (*SI Appendix*, Fig. S2*A*), these comparative analyses suggest that the V_H_3-15-encoded 10E8/DH511 antibodies adopt preconfigured backbone conformations irrespective of lipid-binding status.

Cocrystallization of VRC42.01 Fab, MPER, and 06:0 PA yielded two distinct forms of Fab-MPER complex structure: a lipid-bound structure in space group I222 (VRC42.01-MPER-PA; 3.44 Å resolution) and a lipid-unbound structure in space group C2 (VRC42.01-MPER_unliganded_; 3.13 Å resolution) (Fig. 2*L* and *SI Appendix*, Fig. S1 *D* and *E* and Table S2). The VRC42.01-MPER-PA structure contained one PA molecule in proximity to the Fab CDR H1 and the MPER C-terminus (lipid-binding site 1), indicating biological relevance, and a second PA molecule near the framework regions (FR) H1 and H3 (lipid-binding site 2) located 19.9 Å from the PA molecule located in site 1 (Fig. 2*L* and *SI Appendix*, Fig. S1 *D* and *E*). Lipid-induced structural changes were observed at the CDR H1 and CDR H3 regions in VRC42.01 (0.60 Å RMSD) and PGZL1 (0.38 Å RMSD) between lipid-bound and lipid-free forms when superimposed over MPER (*SI Appendix*, Fig. S3). In contrast, these local structural differences were not observed in 4E10 between its lipid-bound and lipid-free forms, showing greater structural similarity (0.16 Å RMSD).

### DH511.1, DH511.12P, and 10E8 Share a Groove-mediated Lipid-Binding Mode With Distinct CDR Engagement.

Structural and functional studies have shown that the DH511 lineage and 10E8 employ similar MPER- and lipid-binding modes (16). To investigate the molecular basis of this lipid engagement, we compared the DH511.1-MPER-PA and DH511.12P-MPER-PA structures with the previously reported 10E8 structure in complex with the 06:0 PA lipid and the MPER epitope scaffold T117v2, which includes the C-terminal MPER region (residues 671 to 683) renumbered to match the native MPER sequence for structural analysis (Fig. 2 *A*–*I*).

In the 10E8-MPER-PA structure, the 06:0 PA headgroup is accommodated within a compact groove formed by CDR L1 (L28, R29, S30, and Y32) and FR L3 (A66 and G68), with additional stabilization provided by aromatic residues (Y100_E_ of CDR H3 and W680 of MPER) and S100_C_ of CDR H3, positioned along the projected path of the 06:0 PA acyl tail (Fig. 2 *A* and *E*). DH511.1 and DH511.12P utilize a groove-mediated lipid-binding mode similar to 10E8 but with distinct CDR engagements (Fig. 2 *B*–*D* and *F–H*). The DH511.1-MPER-PA, DH511.12P-MPER-PA_one_, and DH511.12P-MPER-PA_two_ structures demonstrated that the 06:0 PA headgroup is positioned within a cavity formed between the base of CDR H3 and CDR L3, contrasting with 10E8, where CDR L3 is exclusively involved in MPER recognition without participating in lipid binding (Fig. 2 *A*–*H* and *SI Appendix*, Fig. S4 *A*–*F*).

Structural superimposition over the MPER region revealed that 10E8 exhibits a unique approach angle relative to the DH511 variants, with the different positioning of CDR L1 and CDR L3 resulting in nonsuperimposable bound lipids and creating antibody-specific grooves (*SI Appendix*, Fig. S2 *D* and *E*). Despite these CDR engagement differences, DH511.1 and DH511.12P employ a headgroup capture strategy analogous to 10E8 (Fig. 2 *E*–*H*). Within the lipid-binding grooves of DH511.1 and DH511.12P, polar residues of CDR L3 (Y92, S93, and S94 in DH511.1; S91, Y92, S93, and S94 in DH511.12P) serve as the core elements for PA headgroup accommodation, functionally equivalent to the polar residues (S30 and Y32) in 10E8 CDR L1.

Furthermore, DH511.1 and DH511.12P create an aromatic cage formed by aromatic residues from CDR H3 (Y100_J_ of DH511.1; F100_D_, Y100_H_, and Y100_I_ of DH511.12P), CDR L1 (Y32), CDR L3 (Y92), and MPER (W680) (Fig. 2 *F*–*I* and *SI Appendix*, Fig. S5). Despite both 10E8 and the DH511 lineage utilizing the same D3-3 gene segment, they employ different reading frames, resulting in distinct CDR H3 amino acid sequences and configurations (*SI Appendix*, Fig. S6 *A* and *C* and Table S1). CDR H3 Y100_H_ of DH511.12P, which appears to contribute to higher lipid occupancy in the double-lipid form, is conserved across plasma cell-derived members, whereas this position is substituted with serine in memory B cell–derived members except for DH511.2 (*SI Appendix*, Fig. S6*A*). This extensive hydrophobic network of the DH511 lineage, which efficiently encapsulates the hydrophobic PA acyl chain, contrasts with the more limited hydrophobic environment of 10E8 and appears to result from differences in CDR H3 D-J junction sequences and V_L_ gene usage (*SI Appendix*, Figs. S5 and S6)

**Fig. 3.**
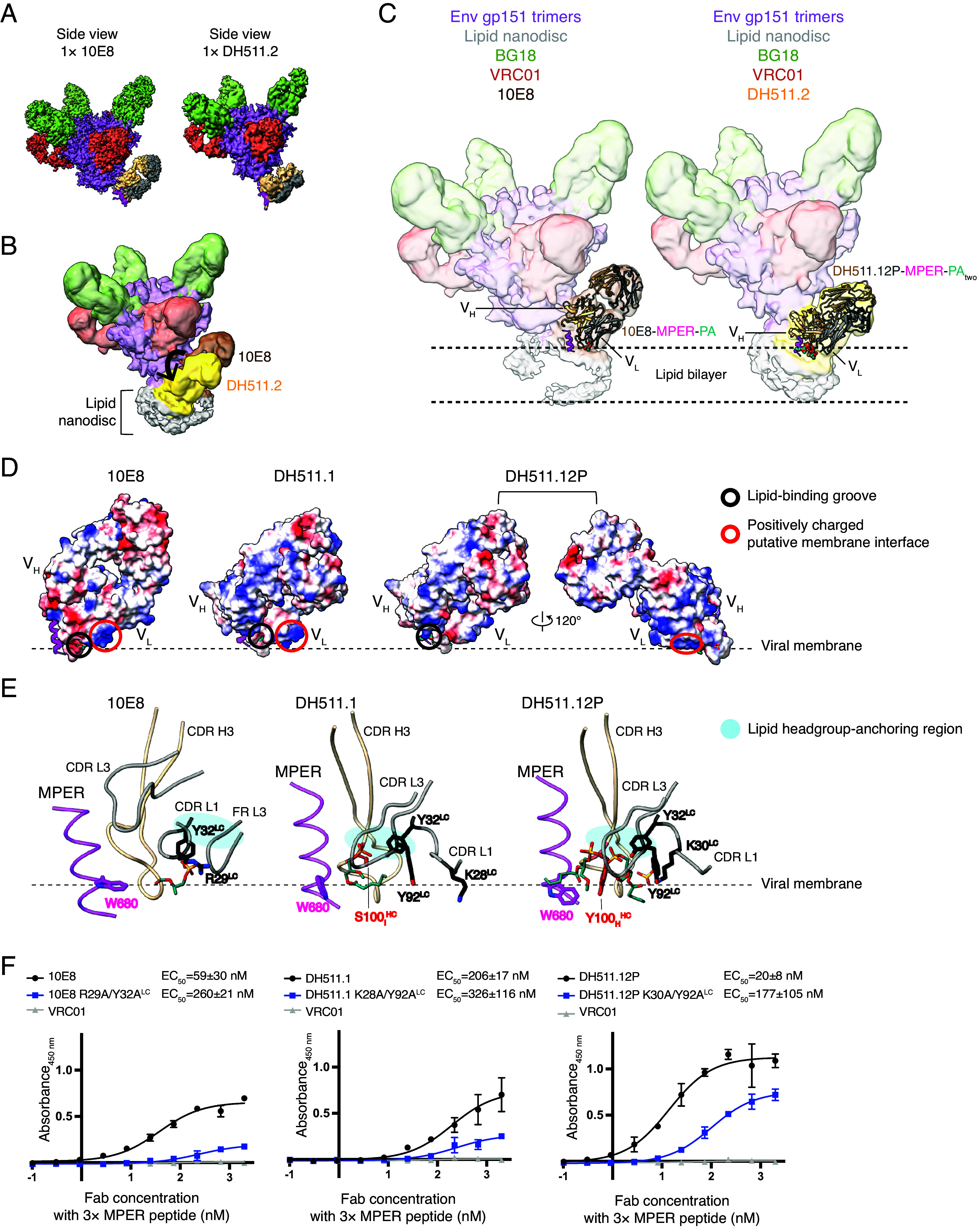
Distinct viral membrane binding approaches and epitope engagement modes of 10E8 and DH511. (*A*–*C*) Cryo-EM reconstructions of 10E8 (EMD-70471) and DH511.2 in complex with nanodisc-assembled Env gp151 trimers. (*A*) Side views of highest-resolution cryo-EM maps of 10E8 (3.5 Å resolution) and DH511.2 (4.5 Å resolution) bound to Env gp151 trimers (ectodomain, purple; MPER, magenta), with BG18 (green) and VRC01 (orange) included to stabilize the Env trimer and facilitate cryo-EM reconstruction. For both 10E8 and DH511.2, heavy and light chains are shown in beige and gray, respectively. (*B*) Gaussian filtered cryo-EM maps of 10E8 (brown; contoured at 0.0226) and DH511.2 (yellow; contoured at 0.0326) superimposed based on the Env ectodomain (purple) in complex with BG18 (green) and VRC01 (orange). Black arrow highlights the rotational shift between 10E8 and DH511.2. (*C*) Gaussian filtered cryo-EM reconstructions of Env bound to 10E8 and DH511.2 with fitted crystal structures of 10E8-MPER-PA (PDB ID: 5T80) and DH511.12P-MPER-PA_two_, respectively. The 06:0 PA molecule is shown with teal sticks with orange phosphate headgroups. Dashed lines indicate the approximate location of viral lipid bilayer. (*D*) Electrostatic potential surfaces (±10 kT/e) of 10E8, DH511.1, and DH511.12P Fabs shown in the cryo-EM-derived orientation in (*C*). The lipid binding groove is indicated in a black circle and the adjacent positively charged putative membrane interface in a red circle. (*E*) Detailed views of the lipid-binding grooves in (D) showing distinct CDR engagement patterns (beige and gray ribbons for heavy and light chains) and key residues for lipid headgroup and membrane anchoring (red and black sticks for heavy and light chains; magenta sticks for MPER). The 06:0 PA molecules are shown as teal sticks with orange phosphate headgroups. (*F*) Functional validation of lipid-anchoring residues through mutational analysis. Individual alanine substitutions were introduced at key lipid-binding residues of Fab light chains (10E8 R29 and Y32; DH511.1 K28 and Y92; DH511.12P K30 and Y92). 14:0 PG binding capacity was then assessed by ELISA in the presence of MPER peptide. The VRC01 protein was used as a negative control. Data represent means ± SD of EC_50_ values from three independent experiments.

To investigate the structural basis for differential epitope engagement, we obtained a cryo-EM reconstruction at 4.5 Å resolution of nanodisc-assembled Env gp151 trimers (Env gp151 ND) in complex with bnAbs DH511.2, BG18, and VRC01 (Fig. 3 *A*–*C* and *SI Appendix*, Fig. S7 and Table S3). Comparison with the previously reported cryo-EM map of Env-BG18-VRC01 complex bound to 10E8 (EMD-70471) revealed distinct binding orientations between the two MPER-targeting antibodies (38). Fitting crystal structures into the cryo-EM density maps showed that, while both antibodies position their V_L_ domains closer to the membrane interface, their V_H_ domains are more distal and dictate their contrasting overall orientations relative to the membrane. DH511 adopts a membrane-facing tilted orientation, while 10E8 maintains an upright orientation toward the Env ectodomain with only its CDR H3 tip extending to the membrane. This tilted binding mode was consistently observed across DH511.1, DH511.2, and DH511.12P in negative-stain and cryo-EM reconstructions (*SI Appendix*, Fig. S8). The resulting angular difference repositions the lipid headgroup-anchoring region from CDR L1-FR L3 in 10E8 to CDR H3 base-CDR L3 in the DH511 lineage, likely reflecting distinct strategies for optimizing engagement of both MPER and viral membrane lipids.

**Fig. 4.**
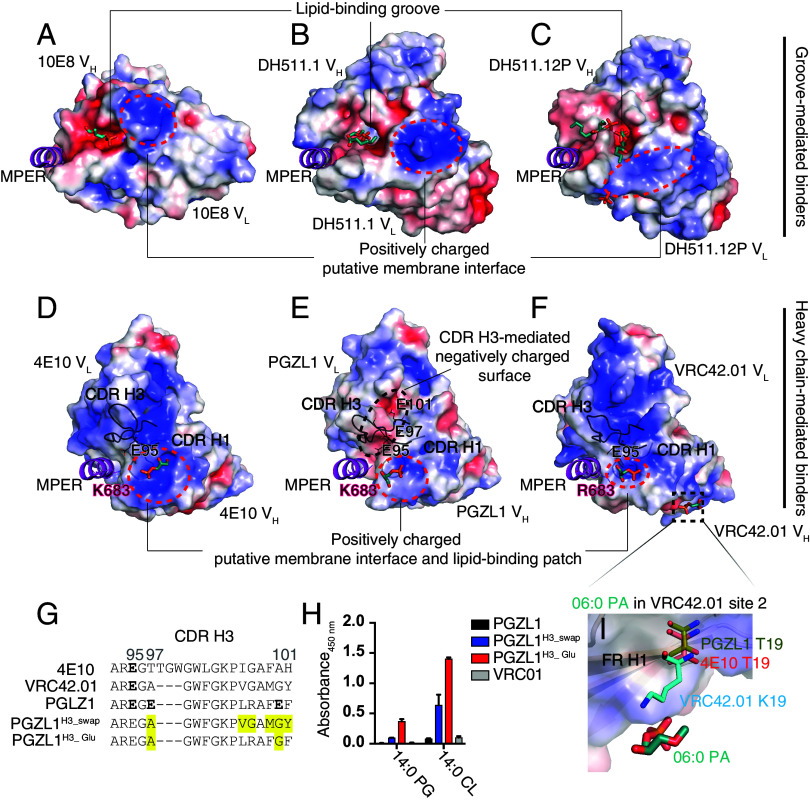
Electrostatic potential surface analyses of HIV-1 MPER bnAbs in complex with MPER peptide and lipids. (*A*–*F*) Electrostatic potential surfaces of the Fab variable domains of 10E8 (*A*), DH511.1 (*B*), DH511.12P (*C*), 4E10 (*D*), PGZL1 (*E*), and VRC42.01 (*F*). Surfaces are colored by electrostatic potential (red, negative; white, neutral; blue, positive) contoured at ±3 kT/e, with MPER peptide (magenta ribbon) and lipid (teal stick with orange phosphate headgroup). The electrostatic potential surface was calculated with APBS. (*G*) CDR H3 sequence alignment of 4E10, VRC42.01, PGZL1, and PGZL1 mutants. Glutamate residues at positions 95, 97, and 101 are in bold letters, and mutated residues are highlighted with yellow backgrounds. (*H*) Effect of the CDR H3-mediated negative charged patch mutations on PGZL1 intrinsic polyreactivity. Binding to 14:0 PG and CL was assessed by ELISA in the absence of MPER peptide. Data are representative of three independent experiments with consistent results. (*I*) Detailed view of the secondary lipid-binding site in K19-mediated positive patch of the VRC42.01-MPER-PA structure. The 06:0 PA molecule (teal stick with orange phosphate headgroup) binds at VRC42.01 FR H1 (beige ribbon) electrostatic surface, with K19 (cyan stick) contrasted against threonine residues at equivalent positions in 4E10 (pink stick) and PGZL1 (olive stick).

Our electrostatic surface analysis revealed that DH511.1 and DH511.12P display positively charged surfaces at the putative membrane interface, located adjacent to the lipid-binding groove (Figs. 3*D* and 4 *A–C*). Both DH511 variants possess two tyrosine residues in the lipid headgroup-anchoring region (Fig. 3*E*): Y32 of CDR L1 (equivalent to 10E8) and Y92 of CDR L3 (DH511-specific), both germline-encoded and conserved across all DH511 lineage members (*SI Appendix*, Fig. S6*B*). When these structures were superimposed over MPER, differences in CDR L1 and CDR L3 positioned 10E8 Y32 between DH511 Y32 and Y92 (*SI Appendix*, Fig. S2*F*). Since Y32 alanine mutants of DH511.1 and DH511.12P could not be expressed, we targeted Y92 alongside basic residues K28 of DH511.1 and K30 of DH511.12P, which were expected to function similarly to 10E8 R29, and generated DH511.1 K28A/Y92A^LC^ and DH511.12P K30A/Y92A^LC^ mutant Fabs. ELISA analysis in the presence of the MPER peptide demonstrated that all three mutants, including 10E8 R29A/Y32A^LC^, exhibited substantially reduced PG binding without significantly affecting MPER binding affinity (Fig. 3*F* and *SI Appendix*, Fig. S9). Given that the analogous 10E8 R29A/Y32A^LC^ mutation substantially reduces neutralization activity while preserving MPER binding (27), disruption of lipid binding at equivalent sites in DH511 may similarly compromise neutralization potency.

Recent multiscale MD simulations demonstrated that the lipid-binding groove of 10E8 accommodates the phosphorylcholine headgroup of phosphatidylcholine and sphingomyelin, which are enriched in the HIV-1 viral envelope (25, 29). To evaluate whether the lipid-binding grooves of the DH511 lineage could also accommodate physiologically relevant phospholipids beyond the PA surrogate, we performed molecular docking of phosphorylcholine (*SI Appendix*, Fig. S10). In the docking models, the phosphate group consistently occupied the crystallographically observed site, while the choline moiety projected into the groove interior without steric clash. Consistent with recent MD simulations of 10E8 (25), this docking model of 10E8 positions the choline moiety adjacent to an array of backbone carbonyl oxygens within CDR L1 and FR L3. In contrast, DH511.1 and DH511.12P appear to employ an aromatic-rich environment in CDR H3 and CDR L3, mediating choline binding through cation–pi interactions (centroid-to-N1 distances of 5.0 to 7.2 Å). These findings support the biological relevance of groove-mediated phosphorylcholine recognition at the HIV-1 viral membrane.

Based on structural and mutational analyses, we propose that, while DH511.1 and DH511.12P employ fundamentally similar groove-mediated membrane recognition strategies as 10E8, they have evolved distinct CDR engagement modes, angular binding approaches, and lipid coordination architectures that optimize their respective neutralization capabilities within the lineage.

### VRC42.01 Shares Heavy Chain-Mediated Lipid-Binding Mode With 4E10 and PGZL1 But Exhibits Distinct Electrostatic Properties.

Previous structural studies demonstrated that 4E10 adopts a perpendicular MPER orientation relative to the viral membrane, similar to 10E8, with the two antibodies rotated approximately 90 degrees relative to one another around their pseudo-dyad axes, positioning 10E8 to interact with the viral membrane primarily through its light chain and CDR H3, while 4E10 interacts through its heavy chain and CDR H3 (Fig. 2) (42, 53).

Comparative analysis of the VRC42.01-MPER-PA structure with the previously reported 4E10-MPER-lipid (PDB ID: 4XC1), in which the bound lipid is sn-glycerol-3-phosphate (G3P), and the PGZL1-MPER-PA (PDB ID: 6O3J) structures revealed similar conformations (RMSD of 0.89 Å and 0.78 Å, respectively) (*SI Appendix*, Fig. S3). The lipid phosphate headgroups occupy the same spatial region when aligned over MPER, confirming a conserved binding mode despite local structural differences across CDR H1, CDR H3, and FR H3 (*SI Appendix*, Fig. S3 *D* and *E*).

In contrast to the groove-mediated lipid binding employed by 10E8 and DH511 antibodies, 4E10, PGZL1, and VRC42.01 utilizes flat surface patches comprising polar residues (particularly threonine or serine at CDR H1 positions 28 and 30) that form direct hydrogen bonds with lipid headgroups (Figs. 2 and 4 and *SI Appendix*, Fig. S4 *G*–*L*). These patches exhibit strong positive electrostatic potentials for accommodating negatively charged lipid phosphate headgroups (Fig. 4 *D*–*F*). In all three antibody-MPER-lipid structures, the MPER K/R683 side chain is positioned at this positively charged interface and contributes to complex stabilization, contrasting the distinct side chain positioning of MPER R683 observed in the VRC42.01-MPER_unliganded_ structure (*SI Appendix*, Fig. S3*F*). While 4E10 exhibits the strongest positive patch at the CDR H1-mediated lipid-binding region, potentially associated with its pronounced polyreactivity, PGZL1 and VRC42.01 show relatively weaker positive charge distributions in the corresponding region. These observations indicate that 4E10, PGZL1, and VRC42, with distinct electrostatic properties, employ direct electrostatic complementarity for lipid engagement, contrasting with the shape complementary binding mode of 10E8 and DH511 antibodies, where positively charged patches are positioned adjacent to, but not directly contacting, the lipid (Fig. 4).

Notably, the positive patch of VRC42.01 extends continuously to the junction between variable and constant heavy domains (Fig. 4*F*). Within this extended region, an additional PA molecule binds to the surface-exposed K19 residue at FR H1, which is V_H_1-69 germline-encoded but substituted with threonine in 4E10 and PGZL1 (Fig. 4*I* and *SI Appendix*, Fig. S11*A*). This lipid-binding site 2 also involves FR H3 residues (Y79 and E81) in lipid headgroup anchoring and hydrogen bonding (Fig. 2*P*). Although a second biologically relevant lipid-binding site has been identified in FR H3 region (A71, D72, R73, and S74 residues) of H4K3, a PGZL1 variant (7), this site does not structurally correspond to the secondary lipid-binding site observed in VRC42.01 (*SI Appendix*, Fig. S12). The biological relevance and potential contribution to polyreactivity of this VRC42.01-specific secondary site require further validation.

### Polyreactivity of PGZL1 Is Modulated By CDR H3-Proximal Negative Charge.

Comparative analysis of electrostatic potentials among 4E10, PGZL1, and VRC42.01 revealed that the PGZL1-MPER-PA structure exhibits a strong negatively charged patch formed by CDR H3 adjacent to the positively charged CDR H1-mediated lipid-binding patch, a feature absent in both 4E10 and VRC42.01 structures (Fig. 4 *D*-*F*). All three antibodies share a common glutamic acid residue, E95, in CDR H3, but PGZL1 specifically possesses glutamic acid residues at positions 97 and 101, while 4E10 and VRC42.01 contain uncharged residues such as alanine, glycine, or threonine at these corresponding positions (Fig. 4*G* and *SI Appendix*, Fig. S12*A*). This substitution pattern enhances the CDR H3-mediated negative charge in PGZL1, creating a more pronounced negatively charged patch compared to the other two antibodies.

To investigate the role of the unique negatively charged patch in lipid binding, we generated two PGZL1 Fab mutants and assessed their lipid-binding properties via ELISA in the absence of MPER (Fig. 4 *G* and *H*). The first mutant involved swapping the entire PGZL1 CDR H3 with the VRC42.01 CDR H3 (PGZL1^H3_swap^), which shares length and sequence similarity, and the second mutant involved replacing only the E97 and E101 residues of PGZL1 CDR H3 with the equivalent A97 and G101 residues from VRC42.01 CDR H3 (PGZL1^H3_ΔGlu^). Both PGZL1 mutants demonstrated markedly increased binding to PG and CL compared to wild-type PGZL1, which showed baseline levels comparable to the negative control (VRC01) (Fig. 4*H*). The binding enhancement was substantially greater for CL than for PG, indicating increased polyreactivity. Notably, the PGZL1^H3_ΔGlu^ mutant, which retained all other residues while substituting only the two specific glutamic acid residues, exhibited a more pronounced increase than the PGZL1^H3_swap^ mutant with extensive residue substitutions.

These findings suggest that PGZL1 maintains a strongly negatively charged patch mediated by CDR H3 in close proximity to the positively charged CDR H1-mediated lipid-binding patch, creating electrostatic repulsion between opposite charges that limits nonspecific lipid reactivity and provides a structural explanation for the reduced polyreactivity previously reported for PGZL1 (7).

## Discussion

This study represents a substantial advance in understanding the complex neutralization mechanisms of bnAbs targeting the HIV-1 MPER. By determining high-resolution structures of MPER and lipid complexes from memory B cell– and plasma cell-derived antibodies of the DH511 lineage and a representative antibody from the VRC42 lineage, we provide a more complete structural framework for understanding the diverse composite epitope recognition strategies across major MPER bnAbs. Furthermore, our findings provide structural blueprints for next-generation HIV vaccine design by elucidating distinct lipid-binding mechanisms and polyreactivity regulation strategies across antibody lineages, offering pathways to circumvent immune tolerance barriers while inducing effective neutralizing responses.

Our structural analyses reveal two fundamentally distinct lipid-binding strategies among MPER bnAbs. 10E8 and DH511 antibodies universally adopt groove-mediated membrane recognition through shape complementarity, forming deep binding grooves that accommodate lipid headgroups, although they differ in the details. In contrast, 4E10, PGZL1, VRC42.01 antibodies employ heavy chain-mediated strategies utilizing flat surface patches that engage lipids primarily through electrostatic complementarity.

Among the groove-mediated binders, 10E8 distinguishes itself by introducing a distinct epitope approach angle evident from the cryo-EM reconstruction, and a distinct CDR engagement pattern compared to DH511, creating a more stringent lipid-binding environment that is narrower and less hydrophobic. This strategy enables 10E8 to accommodate only specific lipids while effectively reducing polyreactivity, reaffirming its promise for vaccine development and therapy. DH511 employs a fundamentally similar polar residue-mediated groove binding as 10E8 but creates a broader and more aromatic-rich environment to accommodate lipid, which may elevate the risk of nonspecific lipid binding. Our negative stain and cryo-EM analyses revealed that each antibody positions its respective lipid-anchoring region in proximity to the membrane interface through distinct angular adjustments.

One of the most significant findings of this study concerns the differential polyreactivity patterns observed within the DH511 lineage. The pronounced cardiolipin-specific polyreactivity enhancement in DH511.1 contrasts with the low polyreactivity of DH511.2 and DH511.12P. Structural and sequence analyses revealed that memory B cell–derived variants predominantly contain serine at the center of the lipid-binding aromatic cage (CDR H3 position 100_I_ in DH511.1), whereas plasma cell-derived variants feature a tyrosine at the corresponding position (CDR H3 position 100_H_ in DH511.12P). This aromatic residue acquisition may enhance lipid coordination and create a more organized binding interface that favors viral envelope-enriched lipids over host membrane lipids. Notably, DH511.2 uniquely retains tyrosine at this position among the memory B cell–derived members and exhibits the highest neutralizing capacity with slightly broader coverage than 10E8 (16). Moreover, the plasma cell-derived DH511.12P demonstrates marginally higher neutralization potency than DH511.2 (16). Collectively, these findings suggest that the low polyreactivity profiles and high neutralization activities observed in DH511.2 and DH511.12P may result from somatic hypermutation and subsequent B cell fate decisions that select against autoreactive properties while maintaining viral neutralization.

The CDR H1-mediated positive patch binding employed by 4E10, PGZL1, and VRC42.01 permits more extensive and flexible lipid binding compared to the groove-mediated binding of 10E8 and DH511, potentially increasing polyreactivity risks. To overcome the high polyreactivity of 4E10, PGZL1 and VRC42.01 appear to adopt strategies including reduction of positive charge at lipid-binding sites, decreased hydrophobicity and flexibility of CDR H3, and introduction of electrostatic repulsion at lipid-binding sites. Both our ELISA results and previous results (17) consistently demonstrate that VRC42.01 exhibits cardiolipin binding, albeit at lower levels than 4E10. While the VRC42 lineage remains a promising vaccine development candidate due to weak polyreactivity at the UCA stage and the ability to achieve broad neutralization breadth with few somatic mutations (17), we suggest that such potential nonspecific binding sites observed in the crystal structure should be considered in vaccine development.

Previous research results clearly demonstrate that strong lipid binding by MPER bnAbs must be accompanied by MPER binding (23–25). The integrated structural analysis of this study provides structural insights into the role of MPER in lipid binding by these antibodies. The MPER W680 residue is commonly found at the lipid-binding interface of 10E8 and DH511, while the MPER K/R683 residue is present at the corresponding interface of 4E10, PGZL1, and VRC42. In DH511.1 and DH511.12P structures, MPER W680 appears to participate directly in stabilizing the lipid hydrophobic tail binding through involvement in aromatic cage formation, showing subtle variations in the W680 indole side-chain orientation between the two antibodies that may enhance lipid accommodation (Fig. 2 *F*–*H*). In 10E8 structures, MPER W680 is positioned toward the lipid tail direction, facilitating hydrophobic tail binding. In contrast, in 4E10/PGZL1/VRC42 structures, the MPER K/R683 side chain commonly extends toward the CDR H1-mediated lipid-binding site and participates in positive charge patch formation, which is supported by previous H4K3 membrane binding models where MPER K683 was proposed to serve as a key structural reference point determining the relative positioning between MPER peptide and the viral membrane (7). These findings collectively demonstrate that distinct MPER residues serve as critical molecular anchors that enable class-specific composite epitope recognition strategies across MPER bnAbs.

Unlike heavy chain-mediated binders such as 4E10, which can directly associate with lipid membranes through electrostatic interactions, groove-mediated MPER bnAbs with lower lipid polyreactivity such as 10E8 and DH511.2 likely engage the viral membrane through a more constrained, sequential mechanism. Although 10E8 possesses intrinsic phospholipid-binding capacity mediated by CDR and framework contacts (25), recent two-step kinetic models suggest that membrane engagement in 10E8 is preferentially initiated by recognition of partially solvent-exposed MPER determinants rather than by spontaneous membrane association (54, 55). In this epitope-assisted docking model, initial MPER encounter may promote subsequent Fab insertion and bilayer stabilization through groove-mediated phospholipid interactions involving lipid headgroups and structural anchors such as W680 (Fig. 2), potentially reinforced by the positively charged putative membrane interface flanking the binding groove (Fig. 3*D*) (56). This sequential mechanism provides a structural basis for the characteristic neutralization breadth and relatively low potency of MPER bnAbs, as the physical constraints imposed by accessing membrane-proximal epitopes limit binding efficiency and kinetic rates (38, 57).

Taken together with recent germline-targeting and human vaccination studies (46, 58), our findings support a model in which the vaccine goal is not elimination of lipid reactivity per se, but its restriction to a peptide-gated, topology-constrained mode of membrane engagement. Our results support a sequential immunization strategy in which germline-targeting immunogens prime low-tolerance-barrier MPER precursor B cells, followed by boosting with more native-like MPER epitope scaffolds (46), and membrane-anchored Env to selectively drive SHM toward clones capable of groove-mediated lipid engagement. A critical consideration is that somatic hypermutation during this process may have inadvertently broadened lipid reactivity; boosting with topologically constrained membrane immunogens, rather than soluble MPER peptides, may impose a requirement for coordinated peptide and membrane recognition, thereby favoring groove-mediated interactions while limiting polyreactivity (27, 54, 55). In this model, phospholipid engagement functions primarily as a secondary anchor that stabilizes the antibody–epitope complex at the membrane surface by slowing dissociation (26, 38, 54). Because such lipid engagement is preferentially reinforced following epitope recognition rather than occurring spontaneously, it need not introduce polyreactivity, allowing potency gains without sacrificing neutralization breadth (27, 55).

The recent success of MPER-based immunogens in inducing precursor bnAb responses in animal models (46), and the demonstration in the HVTN 133 clinical trial that MPER peptide-liposome immunogens can elicit DH1317 lineage antibodies targeting the 2F5 epitope region capable of neutralizing heterologous viruses in humans (58), provide encouraging proof-of-concept for this strategy. The structural insights revealed in this study thus provide a rational basis for guiding affinity maturation toward controlled lipid reactivity that preserves neutralization potency while minimizing autoreactive off-target binding.

## Materials and Methods

### Fab Expression and Purification.

For binding assays, Fab nucleotide sequences encoding heavy and light chains were cloned into AbVec2.0-IGHG1 and AbVec2.1-IGLC2-MscI vectors (Addgene), respectively. Heavy and light chain plasmids were transiently cotransfected into Expi293F cells (Thermo Fisher Scientific) according to the manufacturer’s protocol. Following 7 d of cell culture under controlled conditions (37 °C, 8% CO_2_, 125 rpm), culture supernatants were harvested and purified using CaptureSelect CH1-XL affinity matrix (Thermo Fisher Scientific), followed by size exclusion chromatography on a HiLoad S200 16/600 column (GE Healthcare) equilibrated in Tris-buffered saline (TBS), pH 7.6. Plasmids encoding 10E8, DH511.1, DH511.12P, and PGZL1 Fab mutants were generated using the Q5 site-directed mutagenesis protocol (New England Biolabs), and mutant proteins were obtained using identical procedures as wild-type Fabs.

For crystallization of DH511.1, DH511.12P, and VRC42.01 Fabs, light chain variable region sequences were cloned into a modified AbVec2.1-IGLC2-MscI plasmid engineered to improve the crystallization quality of human Fabs by replacing the human kappa constant domain FG loop (HQGLSSP) with the corresponding rabbit sequence (QGTTS) (59). This modification is restricted to a minor secondary structural element within the constant region, thereby avoiding alterations to the variable domain fold or antigen-binding affinity. Expression and purification of these engineered recombinant Fab proteins were performed using identical procedures as Fabs employed for binding assays.

### Cocrystallization of Fab-MPER Complex With Lipid.

For cocrystallization with Fabs, water-soluble 06:0 PA (830841P, Avanti Polar Lipids) was solubilized to 15 mM in 20 mM sodium acetate, pH 5.5, and the MPER peptide (KKKN_671_WFDITNWLWYIR_683_KKK, where the KKK were added to improve peptide solubility) was synthesized (GenScript) and dissolved to 10 mg/mL in TBS, pH 7.6. To crystallize the Fabs (DH511.1, DH511.12P, and VRC42.01) in complex with MPER and 06:0 PA, purified Fab was concentrated to over 10 mg/mL and incubated with a threefold molar excess of MPER peptide in TBS, pH 7.6, for 30 min at room temperature, then dialyzed using Pur-A-Lyzer™ Mini 6000 (Sigma Aldrich) against 20 mM sodium acetate, pH 5.5, at 4 °C. The resulting complex was mixed with 06:0 PA to a final concentration of 3 mM, and the mixture was incubated for 2 h at 4 °C. Crystal screening was performed at 20 °C using our robotic CrystalMation system (Rigaku) at The Scripps Research Institute in a sitting-drop vapor diffusion format. Crystallization conditions and cryoprotectants are reported in *SI Appendix*, Table S2.

### X-ray Diffraction and Structure Determination.

X-ray diffraction data were collected at synchrotron beamlines specified in the crystal data statistics table (*SI Appendix*, Table S2). Diffraction data were processed using the HKL 2000 program (60). Crystal structures of DH511.1, DH511.12P, and VRC42.01 in complex with the MPER peptide and lipids (or with partial ligand occupancy) were determined by molecular replacement using the respective Fab structures of DH511.1, DH511.12P, and VRC42.01 in complex with the MPER helix (PDB IDs: 5U3J, 5U3N, and 6MTO) as search models (16, 17, 61). The resulting models were refined using the Phenix program and manually adjusted using the Coot program (62, 63). Refinement statistics are listed in *SI Appendix*, Table S2.

### Cryoelectron Microscopy.

Env nanodisc cryo-EM sample preparation followed previously published protocols (38). BG505 MD39.3-based Env gp151 ND construct was first assembled into nanodiscs (64) and complexed with DH511.2, BG18, and VRC01 Fabs. Complex was purified by size-exclusion chromatography and concentrated to 14 mg/mL before vitrification on graphene oxide grids (GO on Quantifoil R2/4, Cu, 200 mesh, SPI Supplies) with 30 s wait time, blot force of 0, and blot time of 2.5 to 3 s. Fluorinated Fos-Choline-8 (Anatrace #F300F) was added to sample immediately prior to freezing to a final concentration of 3 mM. 11,140 micrographs were collected at pixel size 0.718 Å using a Thermo Fisher Scientific Glacios 2 microscope operating at 200 kV and equipped with a Thermo Fisher Scientific Falcon 4i camera using a total dose of 45.0 e^−^/Å^2^. Automated data collection was performed using EPU software (Thermo Fisher Scientific) and images were written in the EER frame format. Micrographs were preprocessed using cryoSPARC Live (65), including motion and CTF correction and were filtered based on CTF fit resolution estimates, resulting in 10,938 micrographs subjected to particle picking. Particles were picked using a combination of blob and template picker jobs. Following multiple rounds of 2D classification, a total of 249,210 particles were extracted at a box size of 600 pixels with Fourier cropping to 448 pixels (resulting in a pixel size of 0.962 Å) to decrease hardware memory requirements. An initial nonuniform 3D reconstruction was performed using an ab initio map as the initial model. Following C3 symmetry expansion, a mask was created over a single density corresponding to DH511.2 Fab. Skip align 3D classification was performed to separate DH511.2-bound versus unbound Env nanodisc. The final stack of 23,082 particles containing DH511.2 Fab density was subjected to Local Refinement with a mask over Env and the Fv regions of each Fab (3× VRC01, 3× BG18, 1× DH511.2), resulting in an estimated global resolution of 4.5 Å (0.143 Fourier shell correlation cutoff). Local resolution was estimated using a mask of the entire complex. Final data collection and processing statistics, and the Electron Microscopy Data Bank accession code are summarized in *SI Appendix*, Table S3. For visualization purpose in Fig. 3 *B* and *C*, Gaussian filters were applied to the cryo-EM maps using UCSF ChimeraX (66) with a SD of 2 voxels.

### Negative-Stain Electron Microscopy.

Env nanodisc in complex with Fab was applied to homemade carbon-coated 400 mesh size copper support grids at 0.04 to 0.06 mg/mL concentration, blotted off with filter paper and stained with 2% (w/v) uranyl formate for 60 s. Data were collected using Leginon automated image collection software on an FEI Tecnai Spirit TEM with an Eagle 4K CCD camera (Thermo Fisher Scientific) (67). The microscope was operated using a voltage of 120 kV and magnification of 52,000X, resulting in a pixel size of 2.06 Å. Data were processed using CryoSPARC and maps visualized in UCSF ChimeraX (66).

### Enzyme-Linked Immunosorbent Assay.

For enzyme-linked immunosorbent assay (ELISA), organic solution-soluble 14:0 PG (840445P, Avanti Polar Lipids) and 14:0 CL (750332P, Avanti Polar Lipids) were dissolved in chloroform:methanol:water (65:25:4) mixture to prepare 10 mM and 5 mM working stocks, respectively.

To quantitatively address the Fab (or Fab mutant) binding to PG lipid in the presence of MPER, PG lipid was further diluted in methanol to a final concentration of 10 μM and coated onto each well of a 96-well plate by allowing methanol evaporation overnight under a fume hood. The following day, the plate was incubated for 2 h at room temperature with blocking solution containing TBS, pH 7.6, with 1% bovine serum albumin. Threefold serially diluted Fab protein with threefold molar excess of MPER peptide was added to the wells and incubated for 2 h at room temperature. After washing with TBS, pH 7.6, wells were sequentially incubated with horseradish peroxidase-conjugated goat antihuman IgG Fab antibody (A56868, Invitrogen) and 3,3’,5,5’-tetramethylbenzidine substrate (ES001, Millipore). After 2 min, the horseradish peroxidase reaction was stopped using sulfuric acid, and plates were read at A450 nm using a microplate spectrophotometer (Tecan Spark). Data were analyzed using the log (agonist) vs. response model in Prism 10 software (GraphPad).

To evaluate MPER-independent polyreactivity of Fab (or Fab mutants) against PG and CL lipids, each well of a 96-well plate was coated with PG or CL (10 μM) and blocked for 2 h at room temperature with the blocking solution. The plate was then incubated with each Fab (2 μM) in the absence of MPER peptide for 2 h at room temperature. Subsequent procedures were identical to those used for quantitative analysis of Fab binding to PG lipid, except that the horseradish peroxidase reaction time was extended to 15 min to maximize the Fab binding signal.

### Biolayer Interferometry.

The binding of each Fab to MPER was evaluated using biolayer interferometry with an Octet Red instrument. For BLI assays, biotinylated MPER peptide (KKKN_671_WFDITNWLWYIR_683_KKK) was synthesized (GenScript) and loaded at 1 μg/mL in 1× kinetics solution containing 1× TBS, pH 7.6, 0.01% bovine serum albumin, and 0.002% Tween 20 onto SA biosensors. Fabs were then associated using twofold serial dilutions starting from 50 μg/mL for 10E8 and its mutant, 80 μg/mL for DH511.1, DH511.12P, and their mutants, and 25 μg/mL for 4E10, PGZL1, and VRC42.01. Experiments were performed at 23 °C, and binding curves were fitted to a 1:1 binding model.

## Supplementary Material

Appendix 01 (PDF)

## Data Availability

X-ray coordinates and structure factors have been deposited in the Protein Data Bank, with the accession codes: 9ZG7 (68) (for DH511.1-MPER-PA), 9ZG8 (69) (for DH511.1_unliganded_), 9ZG9 (70) (for DH511.12P-MPER-PA_one_), 9ZGA (71) (for DH511.12P-MPER-PA_two_), 9ZGB (72) (for VRC42.01-MPER-PA), and 9ZGD (73) (for VRC42.01-MPER_unliganded_). The cryo-EM map of Env gp151 ND in complex with DH511.2, BG18 and VRC01 Fabs has been deposited to the Electron Microscopy Data Bank under access code EMD-74063 (74).
