# Supplementary material for: Structural basis of membrane engagement and polyreactivity control in HIV-1 MPER broadly neutralizing antibodies
Source: Proc Natl Acad Sci U S A. 2026 Jul 14;123(29):e2609827123. doi: 10.1073/pnas.2609827123 (PMC13389661; doi:10.1073/pnas.2609827123)
Supplement: Supplementary file 1 — Appendix 01 (PDF) [file pnas.2609827123.sapp.pdf]

Supporting Information for

Structural basis of membrane engagement and polyreactivity control in HIV-1 MPER broadly neutralizing antibodies

So Yeon Cho[a,c,d], Kimmo Rantalainen[b,c,d], Gabriel Ozorowski[a,c,d], Danny Lu[b,c,d], Ryan Tingle[b,c,d], Wen-Hsin Lee[a,c,d], Andrew B. Ward[a,c,d], William R. Schief[b,c,d,e], Ian A. Wilson[a,c,d,1]

[a]Department of Integrative Structural and Computational Biology, The Scripps Research Institute, La Jolla, CA 92037, USA.
[b]Department of Immunology and Microbiology, The Scripps Research Institute, La Jolla, CA, USA.
[c]IAVI Neutralizing Antibody Center, The Scripps Research Institute, La Jolla, CA 92037, USA.
[d]Center for HIV/AIDS Vaccine Immunology and Immunogen Discovery, The Scripps Research Institute, La Jolla, CA 92037, USA.
[e]Moderna Inc., Cambridge, MA, USA

[1]To whom correspondence may be addressed. Email: wilson@scripps.edu

This PDF file includes:

Figures S1 to S12
Tables S1 to S3
SI References

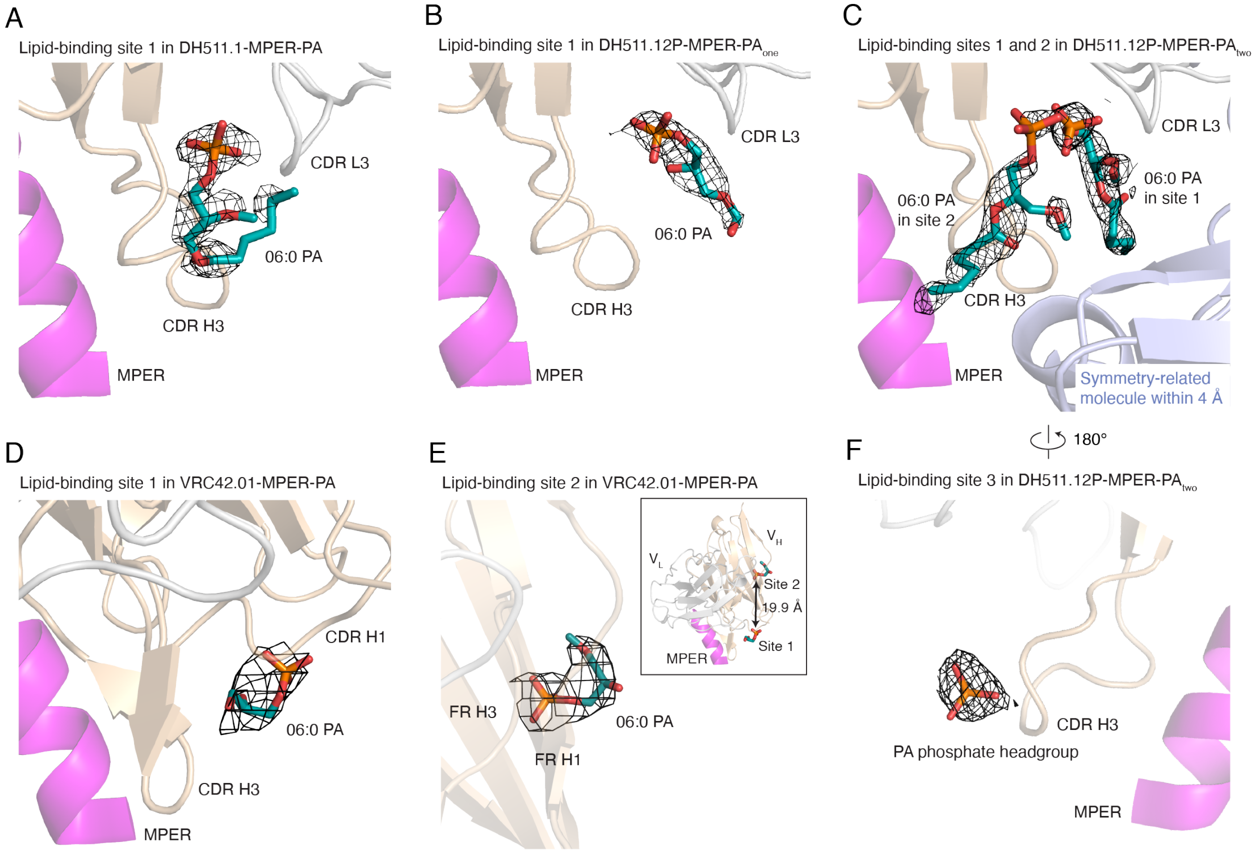

Fig S1. Electron density maps of 06:0 PA binding in DH511.1, DH511.12P, and VRC42.01 crystal structures. The 2Fo-Fc map (1σ level) corresponding to 06:0 PA fragments (teal sticks with orange phosphate headgroup) or isolated phosphate headgroup (orange stick) in the final refined model of DH511.1-MPER-PA (A), DH511.12P-MPER-PA$_{one}$ (B), DH511.12P-MPER-PA$_{two}$ (C, F), and VRC42.01-MPER-PA (D, E) are shown as a black mesh. In the DH511.12P-MPER-PA$_{two}$ structure (C), a symmetry-related molecule (light blue ribbon) is positioned within 4 Å of lipid-binding sites 1 and 2, potentially contributing to the stabilization of the double-lipid occupancy state in the crystal. In (E), solid box insert indicates the relative positions of site 1 and site 2 in VRC42.01.

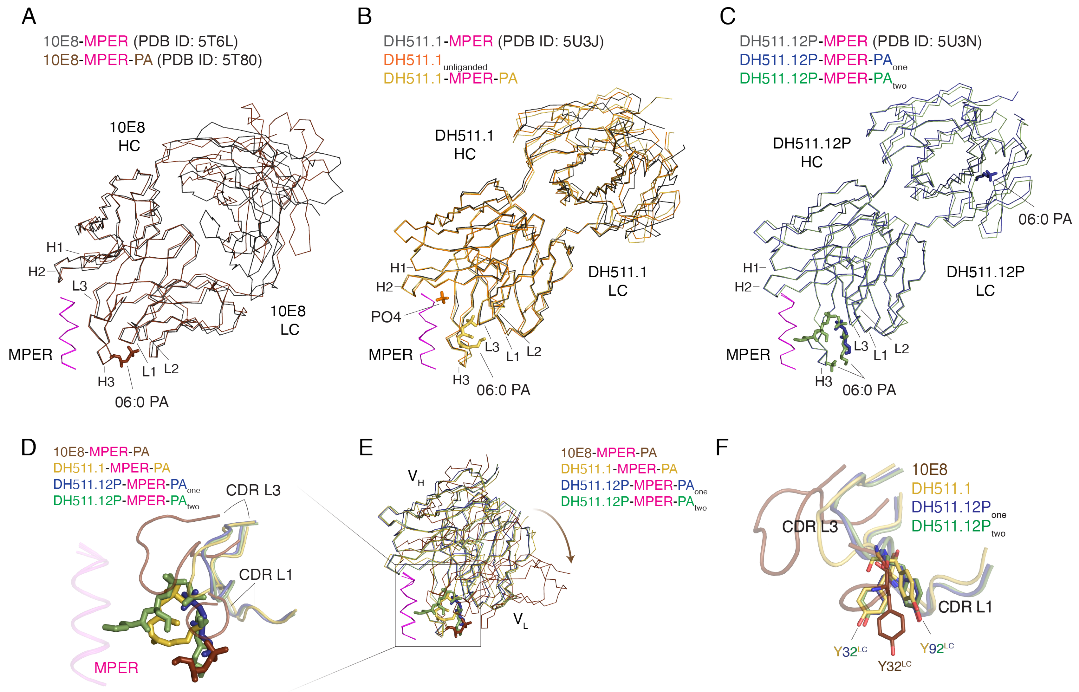

Fig S2. Structural superposition analysis of 10E8, DH511.1, and DH511.12P in complex with MPER and lipids. (A-C) Structural comparison of MPER-bound Fabs (gray Cα traces) from lipid-free structures [10E8-MPER (PDB ID: 5T6L), A; DH511.1-MPER (PDB ID: 5U3J), B; DH511.12P-MPER (PDB ID: 5U3N), C] with corresponding MPER- and lipid-bound Fabs (colored Cα traces) from lipid-containing structures [10E8-MPER-PA (PDB ID: 5T80), A; DH511.1-MPER-PA, B; DH511.12P-MPER-PA$_{one}$ and DH511.12P-MPER-PA$_{two}$, C] or unliganded DH511.1 Fab apo with non-specific phosphate binding derived from the crystallization buffer (DH511.1$_{unliganded}$, B). MPER is shown as magenta Cα traces, and lipids are depicted as colored sticks. (D) Superimposed view of all four structures showing the non-overlapping positions of 06:0 PA molecules and the distinct CDR arrangements, with differences particularly pronounced between 10E8 and DH511. (E) Structural overlay of Fv of 10E8-MPER-PA, DH511.1-MPER-PA, DH511.12P-MPER-PA$_{one}$ and DH511.12P-MPER-PA$_{two}$ superimposed over MPER (magenta Cα trace) with Fab constant regions omitted for clarity. Brown arrow highlights the angular difference between 10E8 and DH511. (1) Structural comparison of tyrosine-mediated lipid anchoring regions in the light chains of 10E8 (brown), DH511.1 (yellow), and DH511.12P in single-lipid form (blue) and in double-lipid form (green). This structural comparison reveals the distinct positioning of tyrosines proximal to the bound lipid between 10E8 and DH511 antibodies

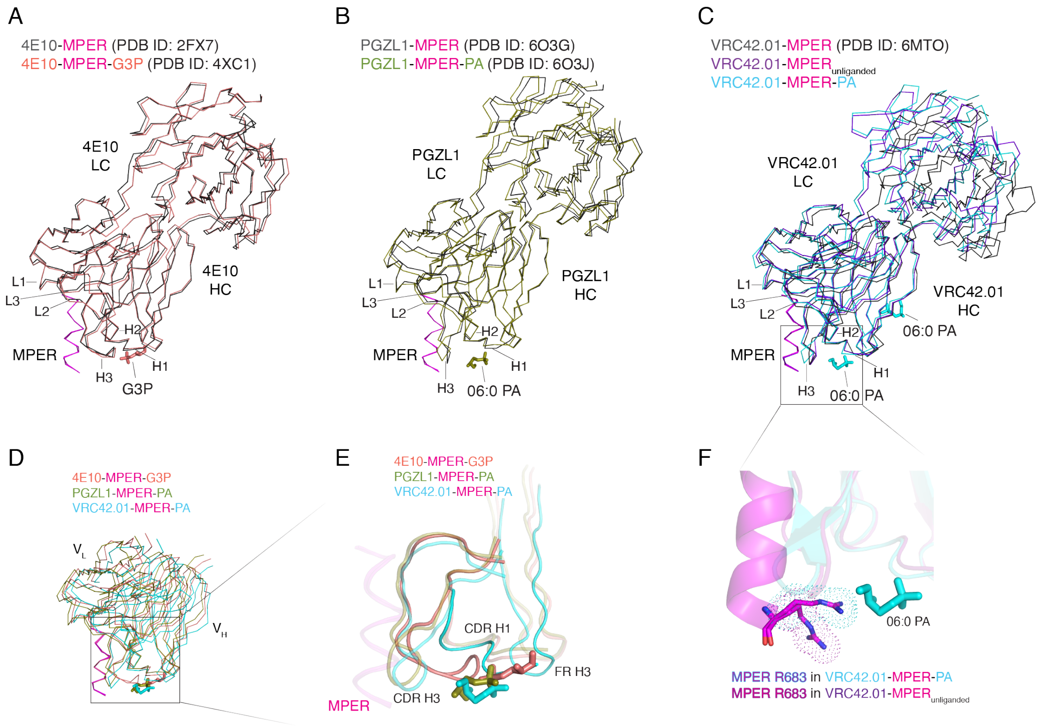

Fig S3. Structural superposition analysis of 4E10, PGZL1, and VRC42.01 in complex with MPER and lipids. (A-C) Structural comparison of MPER-bound Fabs (gray Cα traces) from lipid-free structures [4E10-MPER (PDB ID: 2FX7), A; PGZL1-MPER (PDB ID: 6O3G), B; VRC42.01-MPER (PDB ID: 6MTO), C] with corresponding MPER- and lipid-bound Fabs (colored Cα traces) from lipid-containing structures [4E10-MPER-G3P (PDB ID: 4XC1), A; PGZL1-MPER-PA (PDB ID: 6O3J), B; VRC42.01-MPER-PA, C] or unliganded VRC42.01 Fab-MPER complex ($\text{VRC42.01-MPER}_{\text{unliganded}}$, C). MPER is shown as magenta Cα traces, and lipids are depicted as colored sticks. (D) Structural overlay of Fv of 4E10-MPER-G3P, PGZL1-MPER-PA, and VRC42.01-MPER-PA superimposed over MPER (magenta Cα trace), with Fab constant regions removed for clarity. (E) Superposition showing conserved positioning of 06:0 PA fragments (colored sticks) at lipid-binding site 1 across all three antibodies, despite local structural differences in CDR H1, CDR H3, and FR H3 regions (colored ribbons). (1) Comparison of MPER R683 side chain orientations (magenta sticks) between VRC42.01-MPER-PA (cyan ribbon and sticks) and $\text{VRC42.01-MPER}_{\text{unliganded}}$ (purple ribbon) structures, aligned on MPER (magenta ribbon). The different orientations of the R683 side chain are highlighted with colored dots (cyan for lipid-bound form; purple for lipid-free form).

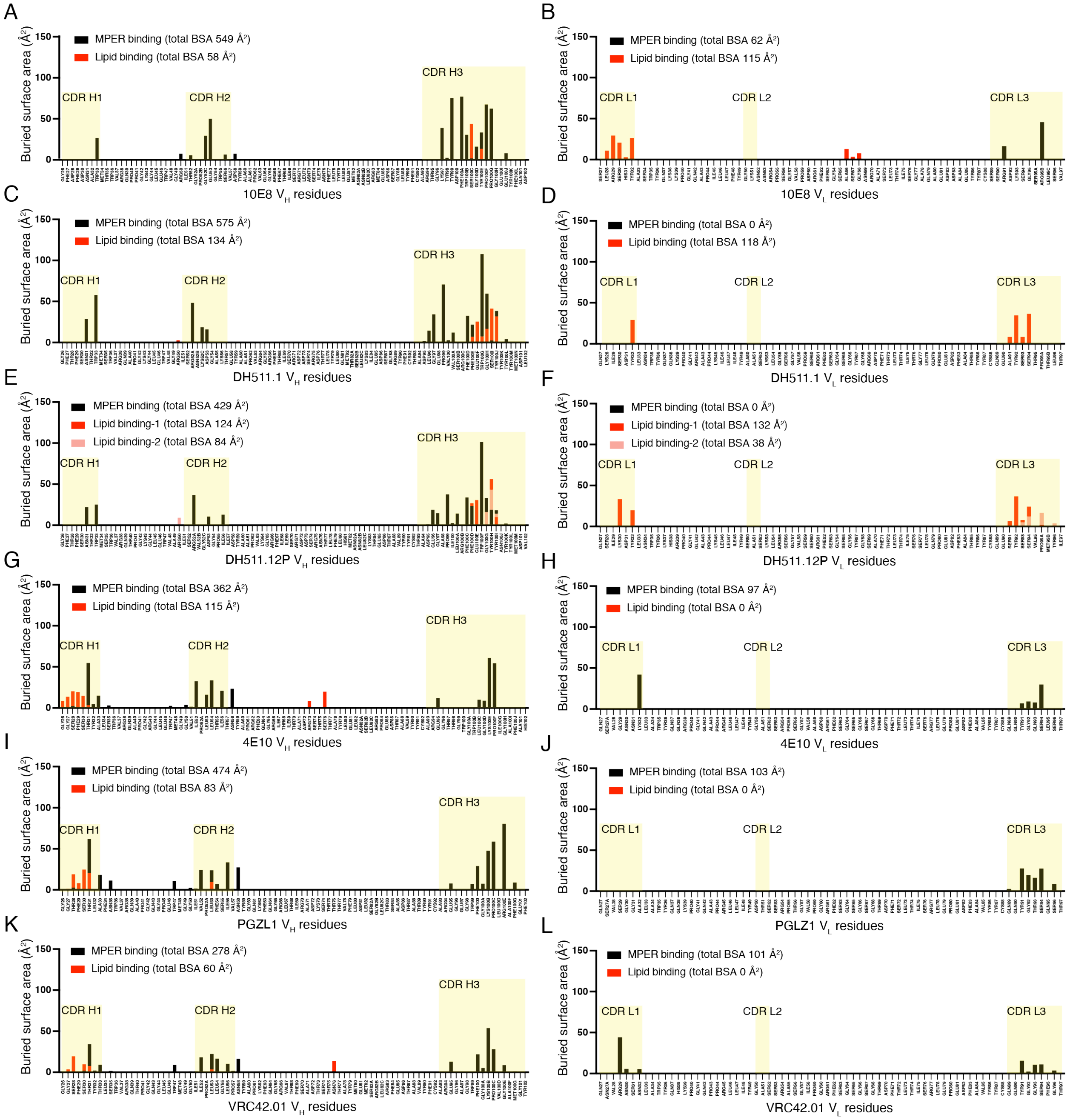

Fig S4. Buried surface area analysis of HIV-1 MPER bnAb Fab-MPER-lipid complexes. Buried surface area (BSA) contributions of individual residues in the variable heavy (V_H) and variable light (V_L) chains are shown for 10E8-MPER-PA (A, B; PDB ID: 5T80), DH511.1-MPER-PA (C, D), DH511.12P-MPER-PA_{two} (E, F), 4E10-MPER-G3P (G, H; PDB ID: 4XC1), PGZL1-MPER-PA (I, J; PDB ID: 6O3J), and VRC42.01-MPER-PA (K, L). MPER-binding residues are depicted as black bars, and lipid-binding residues in site 1 (and also site 2 for DH511.12P) are shown as red or pink bars. CDR regions (Kabat) are highlighted with yellow boxes. FR1 regions, which do not contribute to MPER or lipid binding, are omitted for clarity.

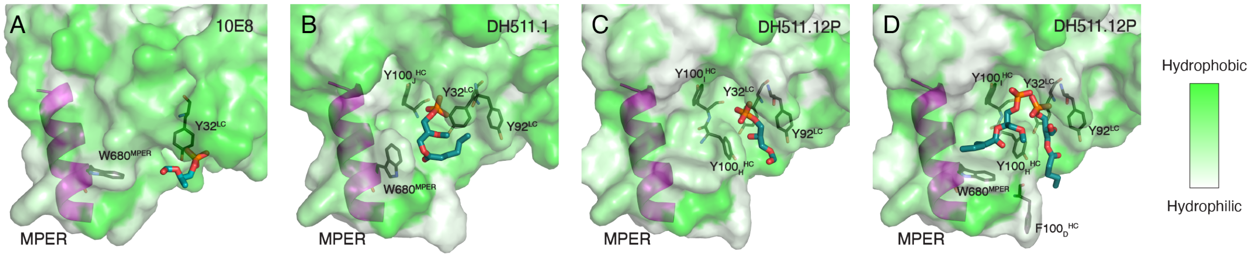

Fig S5. Hydrophobic interface of the lipid-binding site of 10E8, DH511.1 and DH511.12P. (A-D) The Fab-MPER regions in the structures of 10E8-MPER-PA (A), DH511.1-MPER-PA (B), DH511.12P-MPER-PA_{one} (C), and DH511.12P-MPER-PA_{two} (D) are represented as a hydrophobic surface with hydrophobicity-dependent coloring from white (hydrophilic) to green (hydrophobic). The MPER helix is displayed as a magenta ribbon overlaid on the surface. Key aromatic residues involved in lipid interactions (distance ≤4.5 Å) are shown as sticks and labeled. The 06:0 PA lipid fragments are represented as teal sticks with orange phosphate headgroups.

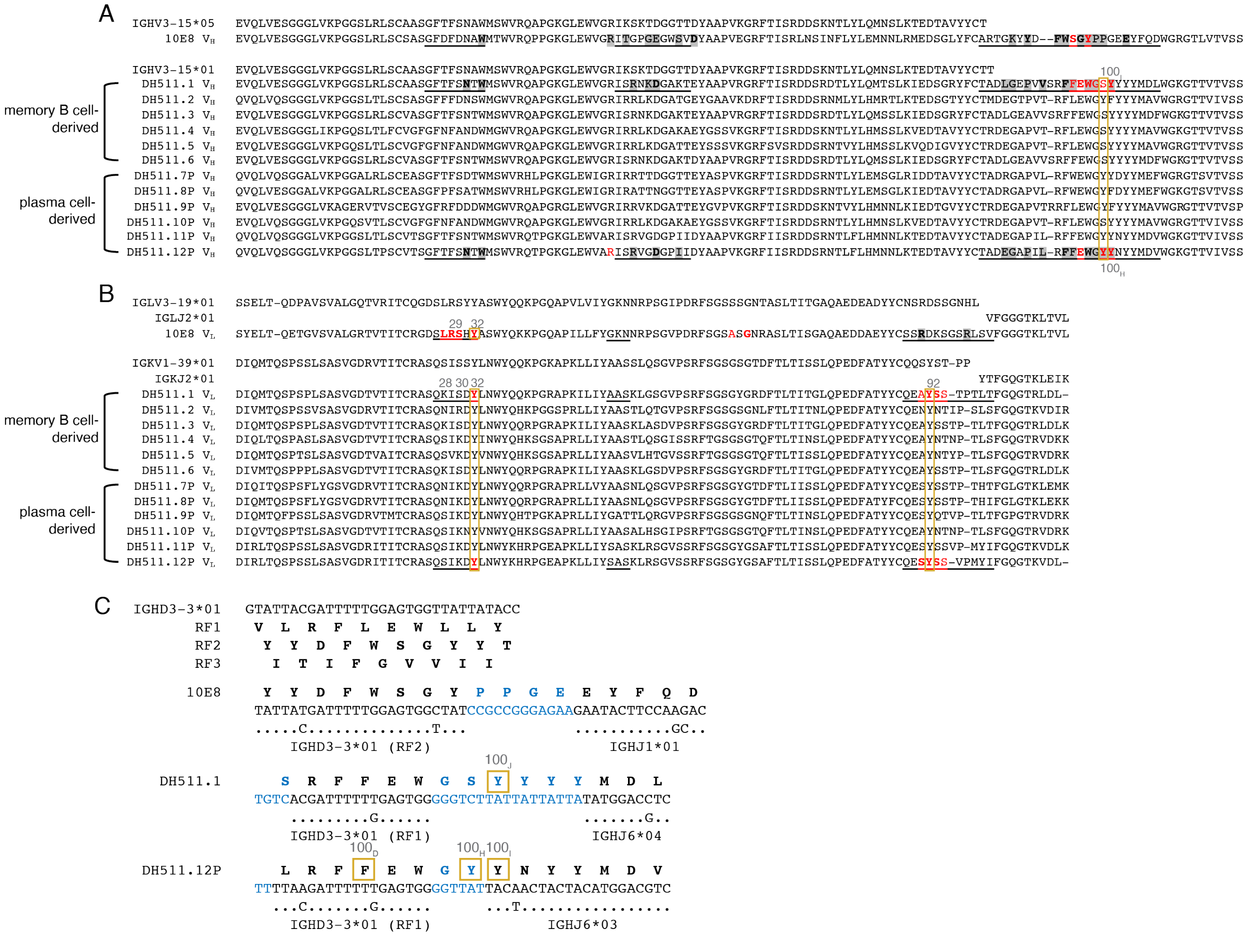

Fig S6. Amino acid sequence alignment of V_H and V_L regions of 10E8 and DH511 lineage members with germline gene sequences. (A) V_H chain sequence alignment showing antibody sequences below their respective germline V (IGHV3-15 alleles) for 10E8 and DH511 lineage members (DH511.1-DH511.6, memory B cell-derived; DH511.7P-DH511.12P, plasma cell-derived). The center residue of the lipid-binding aromatic cage (position 100I in DH511.1, position 100H in DH511.12P) is highlighted with a yellow box. (B) V_L chain sequence alignment showing antibody sequences below their germline V (IGLV3-19*01 or IGKV1-39*01) and J gene segments (IGLJ2*01 or IGKJ2*01). Conserved tyrosine residues involved in the aromatic cage and lipid headgroup anchoring (Y32 and Y92) are highlighted with yellow boxes. In both (A) and (B), residues involved in MPER binding are shown in gray backgrounds, while lipid-binding residues are shown in red letters (sites 1 and 2). Germline-encoded residues among the MPER- and lipid-binding positions are highlighted with bold letters. Key residue positions are indicated by gray numbers above the sequences. CDR regions 1-3 are delineated by black underlines below the sequences from left to right. (C) The germline nucleotide sequence of IGHD3-3*01 is shown at the top with amino acid translations for reading frames 1, 2, and 3 (RF1, RF2, RF3). CDR H3 D-J region sequences of 10E8, DH5111, and DH511.12P are aligned below, showing both amino acid (top line) and nucleotide (bottom line) sequences. 10E8 uses IGHD3-3*01 in RF2 with IGHJ1*01, while DH511.1 and DH511.12P use IGHD3-3*01 in RF1 with IGHJ6*04 or IGHJ6*03, respectively. Residues belonging to the DH511 aromatic cage are highlighted with yellow boxes. Dots indicate nucleotides matching the germline D or J gene sequences. N-region additions and junction-encoded residues are shown in blue.

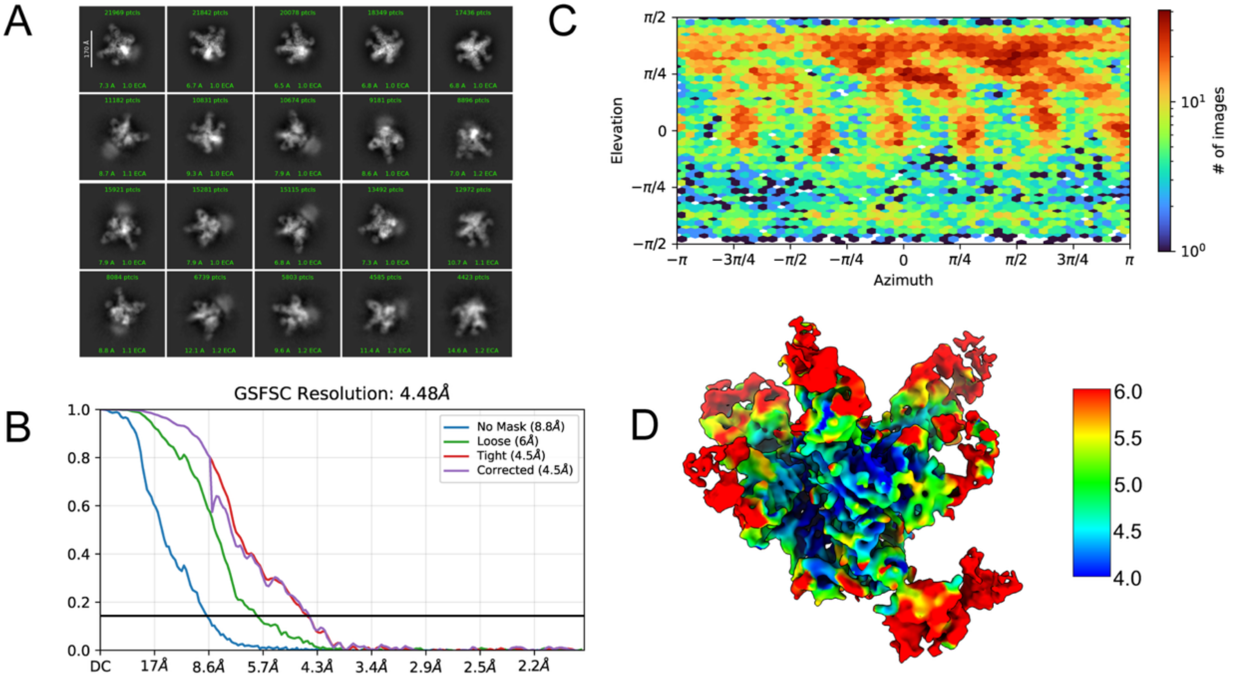

Fig. S7. Cryo-EM data processing statistics. (A) Representative 2D class averages, (B) Fourier shell correlation global resolution estimate (cutoff of 0.143), (C) angular distribution plot of observations, and (D) local resolution estimation (FSC cutoff 0.143) colored according to the resolution (Å) for Env gp151 ND in complex with DH511.2, BG18 and VRC01 Fabs.

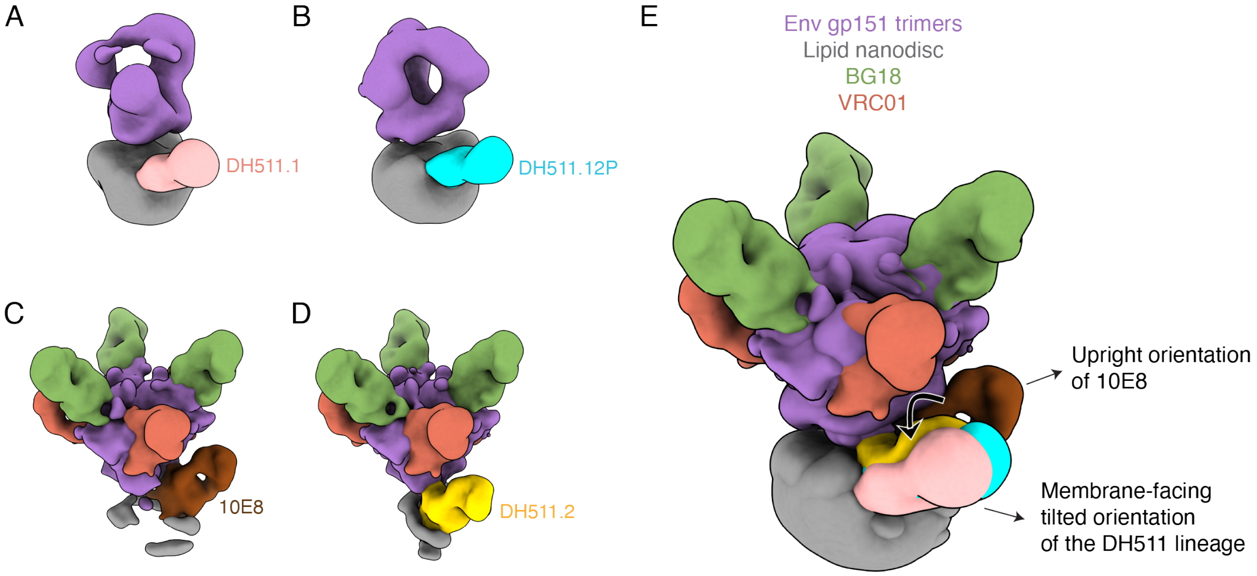

Fig S8. Negative-stain EM of DH511.1 and DH511.12P with nanodisc-assembled Env gp151 trimers.

(A-B) Negative-stain 3D reconstructions of DH511.1 (A) and DH511.12P (B) Fabs (pink and cyan, respectively) bound to Env gp151 trimers (purple) on lipid nanodiscs (gray). (C-D) 15 Å low-pass filtered cryo-EM maps of the trimer-Fab complexes showing 10E8 (EMD-70471, brown) (C) and DH511.2 (yellow) (D) binding orientations for comparison. (E) Overlay of negative-stain and low-pass filtered cryo-EM maps of 10E8 and the DH511 lineage antibodies bound to Env gp151 trimers (purple) on lipid nanodiscs (gray), along with Fabs BG18 in green and VRC01 in orange. Black arrow highlights the distinct binding angles between the upright orientation of 10E8 and the membrane-facing tilted orientation of the DH511 lineage antibodies.

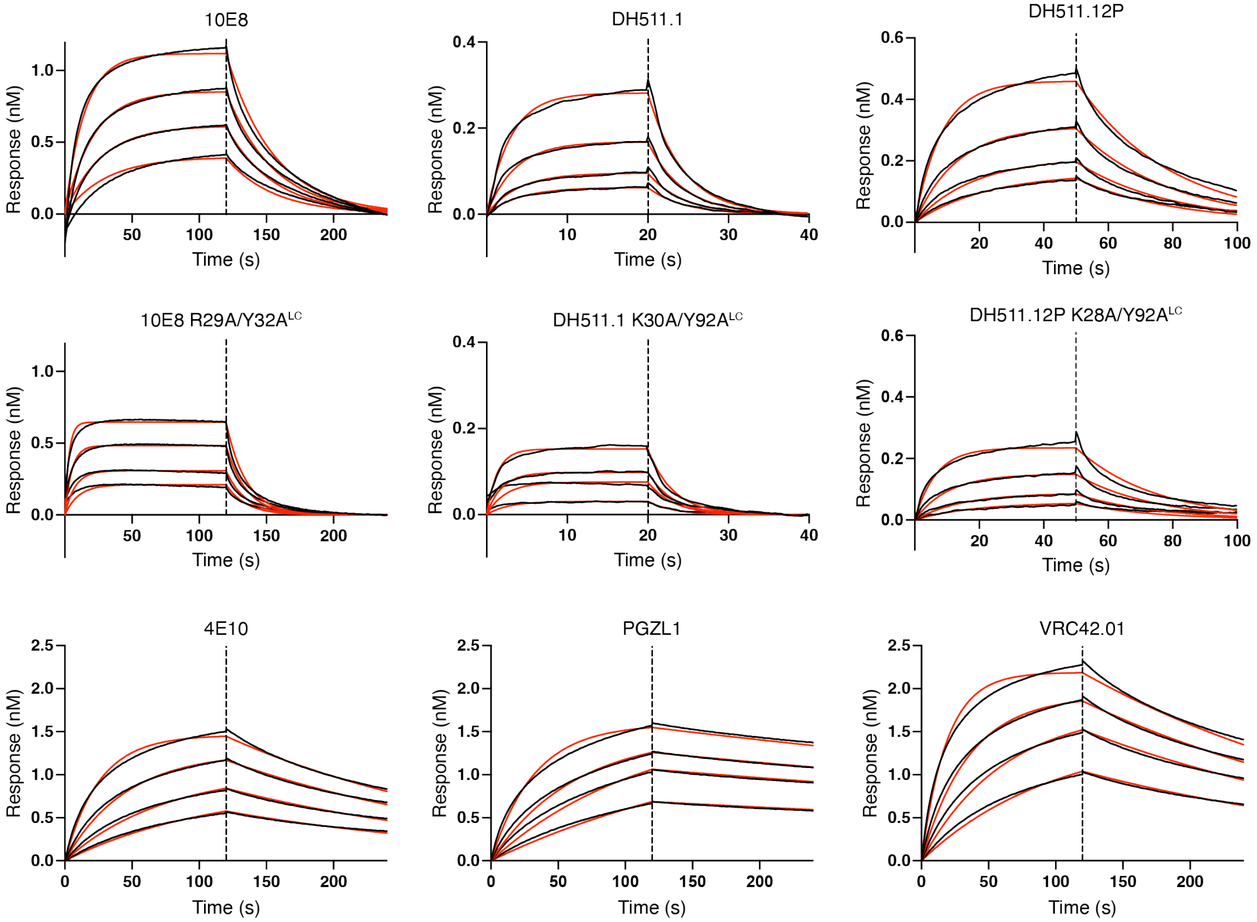

Fab	10E8	10E8 R29A/Y32A^{LC}	DH511.1	DH511.1 K28A/Y92A^{LC}	DH511.12P	DH511.12P K30A/Y92A^{LC}	4E10	PGZL1	VRC42.01
K_D (M)	$7.32*10^{-7}$	$2.63*10^{-7}$	$7.00*10^{-6}$	$2.43*10^{-6}$	$6.67*10^{-7}$	$5.98*10^{-7}$	$7.35*10^{-8}$	$2.10*10^{-8}$	$4.27*10^{-8}$
R^2	0.99	0.99	1.00	0.98	0.99	0.98	1.00	0.99	0.98

Fig S9. Binding of HIV-1 MPER bnAb Fabs and their mutants to MPER peptide by BLI. Bio-layer interferometry sensorgrams showing binding kinetics of various Fab constructs to MPER peptide. Raw experimental data are shown as black lines, and fitted curves are shown as red lines. The vertical dashed line indicates the transition from association to dissociation phase. The binding affinity (K_D) and goodness of fit (R^2) values for each Fab are summarized in the table below the sensorgrams.

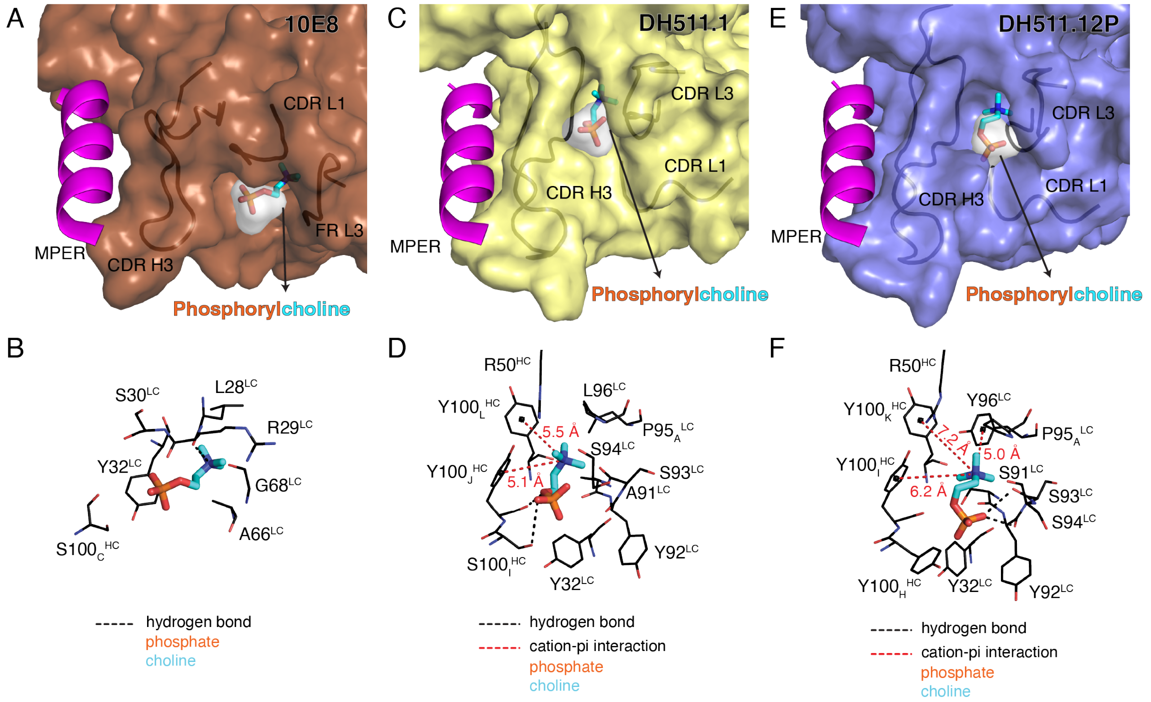

Fig S10. Molecular docking of phosphorylcholine into the lipid-binding grooves of 10E8, DH511.1, and DH511.12P. (A, C, E) Surface representations of the lipid-binding groove regions of 10E8 (A; PDB ID: 5T80), DH511.1 (C; the DH511.1-MPER-PA structure), and DH511.12P (E; the DH511.12P-MPER-PA$_{two}$ structure), with the MPER helix in magenta. The docked phosphorylcholine molecule is shown as sticks (orange and cyan for phosphate and choline groups). The white surface indicates the crystallographically observed phosphate-binding site. CDR regions involved in lipid binding are depicted as ribbons and labeled. (B, D, F) Detailed views of the phosphorylcholine-binding interfaces in 10E8 (B), DH511.1 (D), and DH511.12P (1). Key residues are shown as sticks and labeled using Kabat numbering. Hydrogen bonds and cation-pi interactions are indicated by black and red dashed lines, respectively. In (D) and (1), aromatic ring centroids are shown as black dots.

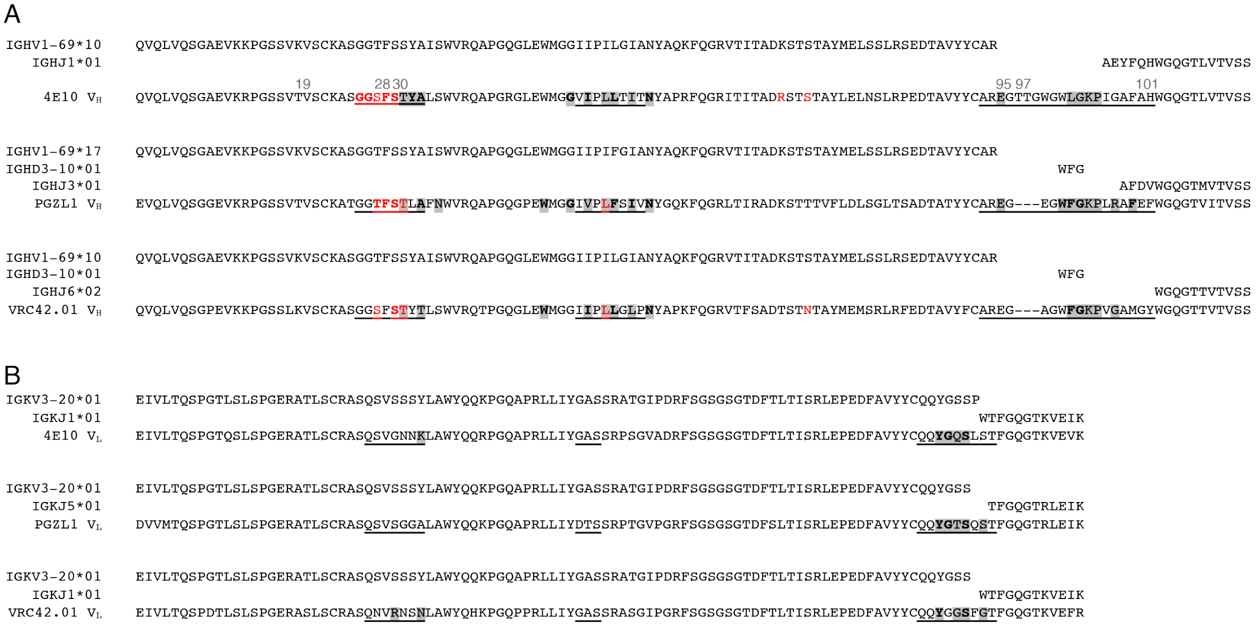

Fig S11. Amino acid sequence alignment of V_H and V_L regions of 4E10, PGZL1 and VRC42.01 with germline gene sequences. (A) V_H chain sequence alignment showing antibody sequences below their respective germline V (IGHV1-69 alleles), D (IGHD3-10*01; not shown for 4E10 due to unavailable nucleotide sequence), and J gene segments (IGHJ1*01, IGHJ3*01 or IGHJ6*02). (B) V_L chain sequence alignment showing antibody sequences below their germline V (IGKV3-20*01) and J gene segments (IGKJ1*01 or IGKJ5*01) In both (A) and (B), residues involved in MPER binding are shown in gray backgrounds, while lipid-binding residues are shown in red letters (site 1). Germline-encoded residues among the MPER- and lipid-binding positions are highlighted with bold letters. Key residue positions are indicated by gray numbers above the sequences. CDR regions 1-3 are delineated by black underlines below the sequences from left to right.

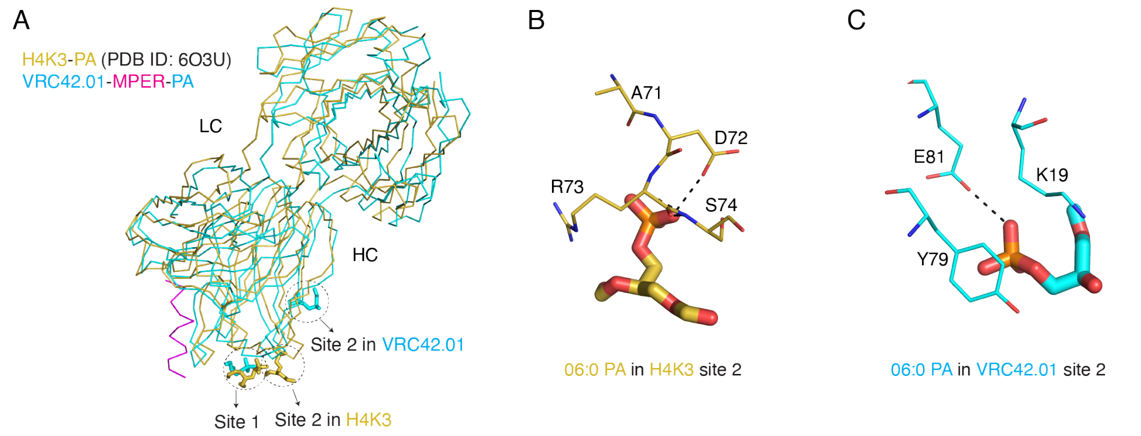

Fig S12. Comparison of lipid-binding sites 1 and 2 between H4K3 and VRC42.01. (A) Structural overlay of VRC42.01-MPER-PA (cyan Cα traces for Fab, magenta Cα traces for MPER, cyan stick for 06:0 PA fragment) with previously determined H4K3 structure in complex with 06:0 PA [H4K3-PA (PDB ID: 6O3U); yellow Cα traces for Fab, yellow stick for 06:0 PA fragment). Site 1 shows roughly superimposable lipid positioning between the two antibodies, while site 2 displays distinct lipid-binding modes. (B, C) Detailed view of lipid-binding site 2 in the H4K3-PA (B) and VRC42.01-MPER-PA (C) structures. Key residues in FR H1 or H3 involved in lipid interactions are shown as sticks. The 06:0 PA fragments at site 2 are displayed as colored sticks corresponding to each antibody structure.

Table S1. Gene usage and somatic hypermutation frequencies of HIV-1 MPER bnAbs.

	Antibody	Putative heavy chain gene alleles			CDR H3 sequence	CDR H3 length (a.a.)	V_H (nt%) mutation frequency	Isotype	Putative light chain gene alleles		CDR L3 length (a.a.)	V_L (nt%) mutation frequency	Major epitope (a.a.)
	2F5	IGHV2-5*02	IGHD3-3*01	IGHJ6*02	RRGPTTSSGVPIARGPVNAMDV	22	12.1	IgG3	IGKV1-13*02	IGKJ4*01	9	11.8	659-671
	10E8	IGHV3-15*05	IGHD3-3*01	IGHJ1*01	ARTGKYYDFWSGYPPGEEYFQD	22	21.5	IgG3	IGLV3-19*01	IGLJ2*01	12	14.8	671-683
memory B cell-derived	DH511.1	IGHV3-15*01	IGHD3-3*01	IGHJ6*04	TADLGEPVVSRFFEWGSYYYYMDL	24	15.7	IgG3	IGKV1-39*01	IGKJ2*01	11	16.3	
	DH511.2	IGHV3-15*01	IGHD4-4*01	IGHJ6*03	TMDEGTPVTRFLEWGYFYYYMAV	23	17.6	IgG3	IGKV1-39*01	IGKJ2*03	11	14	
plasma cell-derived	DH511.12P	IGHV3-15*01	IGHD3-3*01	IGHJ6*03	TADEGAPILRFFEWGYYNYYMDV	23	15.3	IgG	IGKV1-39*01	IGKJ2*01	11	16.4	
	4E10	IGHV1-69*10	IGHD3-10*01	IGHJ1*01	AREGTTGWGWLGKPIGAFAH	20	6.8	IgG3	IGKV3-20*01	IGKJ1*01	9	4.7	671-683
	PGZL1	IGHV1-69*17	IGHD3-10*01	IGHJ3*01	AREGEGWFGKPLRAFEF	17	20.3	IgG1	IGKV3-20*01	IGKJ5*01	9	12.1	
	VRC42.01	IGHV1-69*10	IGHD3-10*01	IGHJ6*02	AREGAGWFGKPVGAMGY	17	10.5	IgG1	IGKV3-20*01	IGKJ1*01	9	5.6	
	LN01	IGHV4-39*07	IGHD3-3*01	IGHJ2*01	VRMPSHGFWSTSFSYWYFDL	20	28	IgG3	IGKV1-39*01	IGKJ1*01	9	27	671-711

Gene usage and V_H/V_L somatic hypermutation frequencies were determined by IgBLAST analysis of available nucleotide sequences for select antibodies (GenBank IDs: JX645769.1 and JX645770.1, 10E8 HC and LC; KY272649.1 and KY272650.1, PGZL1 HC and LC; MH605107.1 and MH605108.1, VRC42.01 HC and LC). For the DH511 lineage members (DH511.1, DH511.2, and DH511.12P), data were extracted from previously reported data (1). For 2F5, 4E10, and LN01, as nucleotide sequence information was not available, amino-acid sequences were analyzed using the IMGT system or data were extracted from previously reported data (2). Color background distinguishes antibody classes (pink, 2F5; blue, V_H3-15-encoded 10E8/DH511; yellow, V_H1-69/V_K3-20/D3-10-encoded 4E10/PGZL1/VRC42; green, LN01).

Table S2. X-ray data collection and refinement statistics

	DH511.1 Fab-MPER(KKK671-683KKK)-06:0 PA	DH511.1 Fab-MPER(KKK671-683KKK)-06:0 PA	DH511.12P Fab-MPER(KKK671-683KKK)-06:0 PA	DH511.12P Fab-MPER(KKK671-683KKK)-06:0 PA	VRC42.01 Fab-MPER(KKK671-683KKK)-06:0 PA	VRC42.01 Fab-MPER(KKK671-683KKK)-06:0 PA
**Reservoir condition**	40% PEG 400, 0.1 M sodium citrate, pH 5.5	20% PEG 8,000, 0.1 M phosphate citrate, pH 4.2, 0.2 M sodium chloride	20% PEG 10,000, 0.1 M Hepes, pH 7.5, 8% ethylene glycol	20% PEG 3,350, 0.2 M potassium iodide	30% PEG 200, 0.1 M Caps, pH 10.5, 0.2 M ammonium sulfate	20% PEG 6,000, 0.1 M citric acid, pH 5.0, 1.0 M lithium chloride
**Cryoprotectant condition**	Included above	Included above	Included above	Included above	Included above	Included above
**Data collection**						
Beamline	NSLS AMX	NSLS AMX	NSLS AMX	NSLS AMX	SSRL 12-1	SSRL 12-1
Wavelength (Å)	0.92015	0.92019	0.92019	0.92019	0.97946	0.97946
Space group	$P2_12_12_1$	$P2_1$	$P2_1$	$P3_121$	I222	C2
Unit cell (a, b, c; Å) (α, β, γ; °)	47.3, 73.1, 142.5 90, 90, 90	39.2, 72.8, 93.0 90, 100.0, 90	76.1, 63.4, 121.4 90, 92.3, 90	73.5, 73.5, 230.4 90, 90, 120	74.4, 128.4, 207.2 90, 90, 90	199.4, 73.5, 44.2 90, 99.4, 90
Resolution (Å) [a]	33.5-2.06 (2.09-2.06)	34.1-1.66 (1.69-1.66)	33.9-2.21 (2.25-2.21)	32.9-1.97 (2.00-1.97)	50.0-3.44 (3.50-3.44)	50.0-3.14 (3.19-3.14)
Unique reflections [a]	31,389 (1500)	60,983 (3056)	57,869 (2906)	52,310 (2553)	13,274 (650)	11,021 (537)
Redundancy [a]	13.6 (13.9)	7.0 (6.3)	7.1 (7.1)	20.4 (20.8)	6.3 (5.8)	4.2 (3.8)
Completeness (%) [a]	99.7 (99.0)	100.0 (99.8)	100.0 (100.0)	99.7 (99.3)	98.4 (98.9)	97.8 (98.0)
$\langle I/\sigma_I \rangle$ [a]	6.0 (1.8)	7.4 (1.0)	6.4 (1.2)	9.4 (1.2)	8.2 (1.6)	10.0 (2.0)
R_{sym} [b] (%) [a]	40.4 (>100)	17.2 (>100)	26.7 (>100)	31.0 (>100)	31.1 (>100)	32.1 (>100)
R_{pim} [b] (%) [a]	11.3 (47.7)	7.0 (76.7)	10.8 (72.3)	7.0 (76.9)	13.3 (49.5)	16.8 (64.7)
$CC_{1/2}$ [c] (%) [a]	99.0 (36.0)	99.6 (31.2)	98.8 (32.0)	99.7 (35.4)	97.8 (62.8)	94.3 (53.6)
**Refinement**						
Resolution (Å)	32.5-2.06	34.1-1.66	33.9-2.21	32.9-1.97	35.0-3.45	35.7-3.10
Reflections (work)	29,814	57,988	54,951	49,810	12,565	10,997
Reflections (test)	1569	2974	2882	2488	668	533
R_{cryst} [d] / R_{free} [e] (%)	21.1/24.7	17.3/20.8	19.0/24.0	22.6/26.4	22.7/26.9	20.9/24.4
**Model composition (asymmetric unit)**						
Fab	1	1	2	1	1	1
peptide	1	-	2	1	1	1
06:0 PA	1	-	3	2	2	-
PO4	n/a	1	n/a	1	n/a	n/a
PEG	1	-	2	3	-	-
Waters	359	672	451	376	-	14
***B*-values**						
Wilson plot (Å²)	22	18	29	28	61	53
Mean isotropic (Å²)	25	22	36	32	61	55
Mean isotropic Fab/solvent (Å²)	26 / 29	22 / 31	39 / 33	35 / 35	67 / -	61 / 40
Mean isotropic MPER peptide (Å²)	45	-	55	41	47	63
Mean isotropic 06:0 PA(s) (Å²)	43	-	70	57	103	-
**RMSD from ideal geometry**						
Bond length (Å)	0.004	0.009	0.012	0.011	0.015	0.005
Bond angle (°)	0.73	1.00	1.14	1.13	1.63	0.66
**Ramachandran statistics (%)** [f]						
Favored	97.6	98.2	96.3	96.8	85.9	95.3
Outliers	0.0	0.0	0.0	0.0	0.2	0.0
**PDB code**	9ZG7	9ZG8	9ZG9	9ZGA	9ZGB	9ZGD

[a] Numbers in parentheses refer to the highest resolution shell.
[b] $R_{sym} = \Sigma_{hkl} \Sigma_i \mid I_{hkl,i} - \langle I_{hkl} \rangle \mid / \Sigma_{hkl} \Sigma_i I_{hkl,i}$ and $R_{pim} = \Sigma_{hkl} (1/(n-1))^{1/2} \Sigma_i \mid I_{hkl,i} - \langle I_{hkl} \rangle \mid / \Sigma_{hkl} \Sigma_i I_{hkl,i}$, where $I_{hkl,i}$ is the scaled intensity of the ith measurement of reflection h, k, l, $\langle I_{hkl} \rangle$ is the average intensity for that reflection, and *n* is the redundancy.
[c] $CC_{1/2}$ = Pearson correlation coefficient between two random half datasets.
[d] $R_{cryst} = \Sigma_{hkl} \mid F_o - F_c \mid / \Sigma_{hkl} \mid F_o \mid \times 100$, where F_o and F_c are the observed and calculated structure factors, respectively.
[e] R_{free} was calculated as for R_{cryst}, but on a test set comprising 5% of the data excluded from refinement.
[f] From MolProbity (3).

Table S3. Cryo-EM data collection, refinement and validation statistics

	BG505 MD39.3 Env gp151 + BG18 Fab + VRC01 Fab + DH511.2 Fab (EMD-74063)
**Data collection and processing**	
Microscope	TFS Glacios
Voltage (keV)	200
Camera	TFS Falcon 4i
Collection mode	Counting
Magnification	190,000x
Pixel size at detector (Å)	0.718
Total electron exposure ($e^{-}/Å^2$)	45
Exposure rate (e^{-}/pixel/sec)	8.043
Number of EER frames	40
Defocus range (μm)	-0.8 to -1.8
Automation software	EPU
Micrographs collected (no.)	11,140
Micrographs used (no.)	10,938
Initial particle images (no.)	249,210
Final particle images (no.)	23,082
Symmetry	C1
Map pixel size (Å)	0.962
Map resolution (masked/unmasked Å)	4.5/8.8
FSC threshold	0.143
Map sharpening *B* factor ($Å^2$)	-82.8
Local map resolution range (Å)	4.0-6.0

SI References

1. L. D. Williams *et al.*, Potent and broad HIV-neutralizing antibodies in memory B cells and plasma. *Sci Immunol* **2**, eaal2200 (2017).
2. L. Zhang *et al.*, An MPER antibody neutralizes HIV-1 using germline features shared among donors. *Nat Commun* **10**, 5389 (2019).
3. C. J. Williams *et al.*, MolProbity: More and better reference data for improved all-atom structure validation. *Protein Sci* **27**, 293-315 (2018).
